# Genomic architecture of phenotypic divergence between two hybridizing plant species along an elevational gradient

**DOI:** 10.1093/aobpla/plw022

**Published:** 2015-08-18

**Authors:** Adrian C. Brennan, Simon J. Hiscock, Richard J. Abbott

**Keywords:** Genetic differentiation, hybridization, phenotypic divergence, QTL architecture, QTL interactions, selection, speciation

## Abstract

Knowledge of the genetic basis of phenotypic divergence between species and how such divergence is caused and maintained is crucial to an understanding of speciation and the generation of biodiversity. The hybrid zone between *Senecio aethnensis* and *S. chrysanthemifolius* on Mount Etna, Sicily, provides a well-studied example of species divergence in response to conditions at different elevations, despite hybridization and gene flow. Here, we investigate the genetic architecture of divergence between these two species using a combination of quantitative trait locus (QTL) mapping and genetic differentiation measures based on genetic marker analysis. A QTL architecture characterized by physical QTL clustering, epistatic interactions between QTLs, and pleiotropy was identified, and is consistent with the presence of divergent QTL complexes resistant to gene flow. A role for divergent selection between species was indicated by significant negative associations between levels of interspecific genetic differentiation at mapped marker gene loci and map distance from QTLs and hybrid incompatibility loci. Within-species selection contributing to interspecific differentiation was evidenced by negative associations between interspecific genetic differentiation and genetic diversity within species. These results show that the two *Senecio* species, while subject to gene flow, maintain divergent genomic regions consistent with local selection within species and selection against hybrids between species which, in turn, contribute to the maintenance of their distinct phenotypic differences.

## Teaser

Hybrid zones that occur across environmental gradients provide excellent opportunities for studying the maintenance of divergent adaptations in the presence of gene flow. They also provide insight into the biodiversity implications of future species contact and hybridization in a changing world. We studied divergent morphology between two *Senecio* (ragwort) species that form a natural hybrid zone with respect to elevation on Mount Etna, Italy, using a quantitative trait locus (QTL) mapping approach. We found signals of divergent selection with increased genetic differentiation close to QTLs. Extensive interactions between QTLs and traits suggested a QTL architecture that is resistant to hybridization.

## Introduction

 Speciation commonly proceeds through genetic divergence between populations that ultimately become reproductively isolated from each other due to intrinsic and/or extrinsic breeding barriers ( Orr and Turelli 2001 ; Coyne and Orr 2004 ; Smadja and Butlin 2011 ; Nosil and Feder 2012 ). Phenotypic trait divergence usually accompanies this process, often as a result of adaptation to different environments ( Nosil 2012 ). Understanding how phenotypic trait divergence evolves between populations and is maintained between hybridizing species requires knowledge of the genetic basis of divergent traits and how selection acts on genes controlling these traits (  Rieseberg *et al.* 2003  ;  Lexer *et al.* 2005  ;  Nosil *et al.* 2009  ; Nosil and Feder 2012 ). 

 Quantitative trait locus (QTL) analysis is a powerful way of analysing the genetic basis of divergent traits between species (  Rieseberg *et al.* 2003  ;  Lexer *et al.* 2005  ;  Bouck *et al.* 2007  ;  Taylor *et al.* 2012  ;  Rogers *et al.* 2013  ). It involves determination of the number and primary effects of QTLs, their genomic locations, the interactions between them (epistasis) and their effects across multiple traits (pleiotropy). The QTL architecture of divergent traits revealed by such analysis is likely to be shaped by divergent selection acting against relatively unfit recombinant hybrid phenotypes (  Bierne *et al.* 2011  ;  Servedio *et al.* 2011  ;  Abbott *et al.* 2013  ; Yeaman 2013 ), especially where divergence between species occurs in the presence of interspecific gene flow ( Via and West 2008 ;  Nosil *et al.* 2009  ; Yeaman and Whitlock 2011 ; Yeaman 2013 ). This selective scenario could favour the evolution of QTL hotspots, epistasis and pleiotropy as effective means of preserving local adaptation despite gene flow (  Whiteley *et al.* 2008  ;  Gagnaire *et al.* 2013  ; Lindtke and Buerkle 2015 ). Alternatively, recombination and break-up of QTL complexes could be reduced by close physical proximity of QTLs ( Yeaman and Whitlock 2011 ;  Jones *et al.* 2012  ; Yeaman 2013 ) or recombination ‘coldspots’ such as near centromeres or chromosomal rearrangements (  Turner *et al.* 2005  ; Kirkpatrick and Barton 2006 ; Lowry and Willis 2010 ; Twyford and Friedman 2015 ). 

 Complementary insights into the relationship between QTL architecture and divergent selection can be obtained by investigating genetic diversity and differentiation among mapped molecular marker loci ( Rogers and Bernatchez 2007 ; Stinchcombe and Hoekstra 2008 ;  Gompert *et al.* 2012  ;  Renaut *et al.* 2012  ;  Strasburg *et al.* 2012  ; Cruikshank and Hahn 2014 ). Heterogeneous differentiation across the genome is expected to result from divergent selection in the presence of gene flow ( Wu 2001 ; Feder and Nosil 2010 ) and has been reported in several studies of ecologically divergent pairs of taxa (  Turner *et al.* 2005  ; Rogers and Bernatchez 2007 ; Via and West 2008 ). However, such patterns of differentiation can be highly dependent on the biology and demographic histories of the focal taxa (  Jones *et al.* 2012  ;  Renaut *et al.* 2012  ), and their assessment must take account of genetic diversity both within and between focal taxa ( Cruikshank and Hahn 2014 ). 

 Here, we present a quantitative genetic analysis of divergent traits between two diploid (2 *n* = 20), short-lived perennial, self-incompatible, herbaceous species of *Senecio* (Asteraceae), *S. aethnensis* and *S. chrysanthemifolius* , which grow at elevations above 2000 m and below 1000 m, respectively, on Mount Etna, Sicily. Whereas *S. aethnensis* produces large flower heads (capitula) and fruits, and entire (spathulate) leaves, *S. chrysanthemifolius* has smaller flower heads and fruits, and highly dissected (pinnatisect) leaves. The two species hybridize and form a hybrid zone at intermediate elevations on Mount Etna ( James and Abbott 2005 ; Abbott and Brennan 2014 ). Although connected by hybrid populations, some barriers to interspecific gene flow are apparent in the field. For example, flowering times only partially overlap, with *S. chrysanthemifolius* flowering 6 weeks earlier (April–June) than *S. aethnensis* (July–September) (authors’ personal observation). A previous analysis of the hybrid zone showed that leaf shape, flower head structure and fruit structure exhibited steeper clines and/or shifts in cline position relative to a molecular genetic cline (  Brennan *et al.* 2009  ). This was attributed to both intrinsic and extrinsic environmental selection against hybrids. 

 An improved understanding of the level of genetic divergence between the two species and the importance of selection in driving genomic divergence recently came from a comparison of their transcriptomes (  Chapman *et al.* 2013  ). This showed that genome-wide genetic differentiation between the species was low, with only 2.25 % of 8854 loci tested having been subject to divergent selection. Genetic maps for the two *Senecio* species based on segregation of molecular markers in F _2_ mapping families (  Brennan *et al.* 2014  ;  Chapman *et al.* 2016  ) indicated that large genomic rearrangements were not a cause of reduced fitness in hybrids. However, many markers (∼27 % of 127 maker loci tested,  Brennan *et al.* 2014  ) exhibited significant transmission ratio distortion (TRD) in the F _2_ family and clusters of TRD loci (TRDLs) were distributed across multiple linkage groups. This frequency of TRD was similar to that found in other crossing studies involving distinct ‘species’ (e.g. 49 and 33 % in *Mimulus* and *Iris* , respectively,  Fishman *et al.* 2001  ;  Taylor *et al.* 2012  ). Such extensive genomic incompatibility between the two species would be expected to affect the genetic structure of the hybrid zone on Mount Etna by limiting interspecific gene flow and promoting divergence across the genome.  Chapman *et al.* (2016)  further showed that loci exhibiting significant sequence or expression differentiation between the two species had a clustered distribution when placed on the map and several QTLs for species phenotypic differences coincided with these regions. 

 Here, we investigate further the genetic architecture of phenotypic trait differences and associated divergent selection acting on *S. aethnensis* and *S. chrysanthemifolius* by performing a QTL analysis of multiple quantitative traits that distinguish the two species. Our analysis examined additional traits and a larger mapping family relative to the recent study by  Chapman *et al.* (2016)  , albeit with a reduced number of molecular marker loci. Our study aimed to determine the number and genomic locations of QTLs of relatively large effect controlling phenotypic differences and the extent of epistatic and pleiotropic effects of QTLs that could limit introgression between the two species in the wild. We also conducted genetic differentiation outlier tests on mapped molecular markers in the two species to identify loci under divergent selection and test for associations between outlier loci and QTLs. In addition, we tested whether previously identified hybrid incompatibilities are associated with either QTLs for species differences or highly divergent loci as would be expected under divergent selection. 

## Methods

### Samples

 An F _2_ mapping family (F _2_ AC) of a reciprocal cross between two cross-compatible F _1_ progeny derived from a reciprocal cross between *S. aethnensis* (A) and *S. chrysanthemifolius* (C) was produced as described in  Brennan *et al.* (2014)  and used for QTL analysis. This family consisted of 100 individuals of known parental cytotype. For tests of selection based on genetic differentiation, seed was collected from two wild populations of *S. aethnensis* and three of *S. chrysanthemifolius* representing the elevational extremes of each species’ range and also the source locations of the mapping family parents (NIC1 and PIC1) ** [see Supporting Information—Table S1 ] ** . Forty-two plants of each species, each representing a separately sampled maternal individual, were raised from this seed in a glasshouse at the same time and under the same conditions as F _2_ AC individuals. 

### Phenotype measurement

 Twenty-five traits were measured on F _2_ AC parents and progeny, and also wild sampled individuals (see  Brennan *et al.* 2009  for a description of traits measured). Extreme outlier values >3 standard deviations from the mean were removed from the datasets for progeny and wild samples of each species prior to analysis. Trait summary statistics were calculated and comparisons between wild sampled *S. aethnensis* , wild sampled *S. chrysanthemifolius* and the F _2_ AC mapping family were made using one-way analyses of variance and Mann–Whitney tests. Three traits—capitulum length, ray floret number and selfing rate—were dropped from further analysis after preliminary data exploration found that they showed extreme distributions that could not be satisfactorily resolved with data transformations. Remaining trait measurements were not transformed to become normally distributed before QTL analysis because (i) the expected density distributions of traits with additive effects contributed by multiple loci are not necessarily normally distributed, (ii) the significance of QTL logarithm of odds (LOD) scores can be adequately assessed with data permutation and (iii) estimated sizes of QTL effects are more directly interpretable based on untransformed data ( Churchill and Doerge 1994 ). Cross direction did not significantly influence any trait mean, so this was not required as a cofactor for QTL analysis. Independence between measured traits was examined using paired-trait Spearman correlations, and tests of their significance were performed separately for wild sampled *S. aethnensis* , wild sampled *S. chrysanthemifolius* and the F _2_ AC mapping family progeny leading to a subset of 13 highly independent traits being retained for QTL analysis. All tests were performed using R v2.13 software ( R Development Core Team 2011 ). 

### DNA isolation and genotyping

 DNA was extracted from each plant using the method described by  Brennan *et al.* (2009)  . Plants were genotyped across 127 marker loci comprising 77 amplified fragment length polymorphisms (AFLPs), 8 simple sequence repeats (SSRs) and 42 expressed sequence tag (EST)-SSRs and indel molecular markers as described by  Brennan *et al.* (2014)  . For ∼10 % of plants (randomly chosen), two independent DNA extracts were made to test for genotyping reliability. 

### Genetic mapping

 A genetic map was constructed from the segregation of genetic markers in the F _2_ AC mapping family as described in  Brennan *et al.* (2014)  and Supplementary information . Genotype uncertainty due to scoring of dominant markers was accounted for by using the MapMaker genotype classes C (not a homozygote for the first parental allele) and D (not a homozygote for the second parental allele;  Lincoln *et al.* 1993  ). The genetic map comprised 10 independent linkage groups with a total length of ∼400 cM. Transmission ratio distortion affected ∼27 % of mapped markers that were clustered into nine TRDLs. Sixty-five mapped loci were included in the QTL analysis after removing 39 loci that did not show F _2_ -like allelic segregation (i.e. each parent had an allele in common) and 23 loci that were located <0.5 cM from the nearest neighbouring marker and which therefore added little extra QTL mapping power. 

### Quantitative trait locus mapping and analysis

 We analysed the data in the form of individual differences from the combined species mean, with the sign altered so that individuals that were more similar to *S. aethnensis* or *S. chrysanthemifolius* mean values were positive and negative, respectively. This data transformation preserved effect sizes in original units but had the added advantage of standardizing effect directions according to parental species across all traits. Comparisons with untransformed data showed that LOD scores (base 10 logarithm of odds) were largely unaffected by the transformation. Multiple interval mapping (MIM) was used to identify QTLs because this method has the advantage of simultaneously accounting for multiple QTLs and their interactions (  Kao *et al* . 1999  ). Multiple interval mapping was performed with QTL cartographer v2.5.10 (  Wang *et al.* 2011  ) using forward regression with a scanning interval of 3 cM and Bayesian information criterion (BIC-M0) model selection to determine the inclusion of extra QTL or QTL interaction parameters. Initial MIM models were then refined by testing indicated QTLs for significance according to BIC and adding additional QTLs until no further significant model improvement was achieved. Epistatic QTL interactions were also included if BIC was significantly improved. For comparison with MIM, composite interval mapping (CIM; Zeng 1994 ), a widely used QTL mapping method, was also performed and results obtained from this analysis, which did not differ greatly from those obtained with MIM, are presented in Supplementary information . The potential for TRDLs to influence the QTL results was tested using Spearman rank correlation tests of marker distance to nearest QTL peak against marker *χ* ^2^ test values for segregation distortion of genotypes, heterozygotes and parental alleles. 

 Multiple trait CIM (MtCIM) simultaneously analyses multiple trait data and can distinguish between linked QTLs and a single QTL affecting more than one trait through pleiotropy (MtCIM; Jiang and Zeng 1995 ). Multiple trait CIM analysis was performed using a scanning interval of 3 cM and automatic model selection using forward regression with five cofactor loci outside the test interval window of 10 cM. Significance of QTL LOD scores was tested with 1000 permutations of trait values ( Churchill and Doerge 1994 ). A complementary test of the extent to which QTLs for different traits occupied the same genomic regions applied the ‘sampling without replacement’ method ( Paterson 2002 ). Because the traits examined in this QTL dataset were selected to minimize covariance between them, spurious patterns of QTL coincidence generated by covariance were also assumed to be minimized, avoiding the need for additional statistical correction (  Breitling *et al.* 2008  ). To perform the ‘sampling without replacement’ test, the genetic map was divided into smaller intervals of equal size corresponding to the mean QTL 2-LOD cM confidence interval of 16.5 cM with intervals chosen to be centred over each linkage group. This level of subdivision of the genetic map generates an optimal proportion of intervals occupied by a QTL for the purposes of this test ( Paterson 2002 ), but the effect of using smaller interval sizes was also tested by repeating the test with 2, 4, 6, 8, 10, 12 and 14 cM interval sizes. A binary matrix describing the presence or absence of QTLs for each trait within intervals was constructed and for each pair of traits, the probability of coincidence ( *p* ) was tested according to:

$$p=\left(\begin{array}{c} l\\ m \end{array}\right)\left(\begin{array}{c} n-l\\ s-m \end{array}\right)/\left(\begin{array}{c} n\\ s \end{array}\right)$$

where *n* is the number of intervals compared, *l* and *s* are the number of QTL intervals present in the samples with larger and smaller QTL counts, respectively, and *m* is the number of paired QTL interval matches present. To test whether QTL coincidence was greater than the null hypothesis of a random distribution of QTLs across the genetic map, the observed mean probability of QTL coincidence across paired-trait comparisons was compared against the distribution obtained from 1000 random permutations of QTL locations. The coincidence between TRDLs and QTLs was also investigated by including TRDL data in this analysis. 

### Genetic diversity analysis

 Summary population genetic statistics were estimated for all mapped markers genotyped in wild samples of *S. aethnensis* and *S. chrysanthemifolius* . The population genetics software used included: Arlequin ( Excoffier and Lischer 2010 ), GenAlEx v6.1 ( Peakall and Smouse 2006 ) and HPrare ( Kalinowsky 2005 ). The estimated statistics for AFLP and other dominantly scored markers were band presence frequency ( *p* ; assuming Hardy–Weinberg equilibrium), effective number of alleles (Ne), unbiased heterozygosity (UHe), allelic richness (Ar), private allelic richness (pAr), genetic differentiation among species ( *F*_ST_ ) and genotypic differentiation ( *Φ*_PT_ ). The same statistics, excluding *p* but including the minor allele frequency (MAF) and inbreeding coefficient ( *F*_IS_ ), were calculated for codominantly scored markers. 

 Patterns of differentiation across loci were investigated to detect both strongly and weakly differentiated outlier loci using BayeScan ( Foll and Gaggiotti 2008 ), which employs Bayesian methods to estimate locus-specific differentiation and to evaluate its probability relative to population-level differentiation. Default starting parameter settings were used, except for a Monte Carlo Markov Chain size of 10 000, thinning interval of 50, ten pilot runs of 10 000 and an additional burn-in of 1 00 000. Outlier loci were identified based on log _10_ Bayes Factors values greater than one. Outlier analysis was performed with individuals classified according to both species and population. Initial runs suggested that loci with very low MAF were over-represented among outliers. To overcome this problem, only those loci with MAF >0.05 were included in final differentiation analyses, which were conducted separately on datasets comprising 64 codominant loci and 132 dominant loci. 

 The presence of ‘genomic islands’ of divergence was investigated by testing the genomic clustering of outlier markers with binomial tests that the observed number of neighbouring pairs of significantly selected loci was greater than the expected number of neighbouring paired selected loci given by the square of the observed frequency of selected loci. Genetic differentiation, measured as both *F*_ST_ and *Φ*_PT_ , was tested for an association with the genetic map distance to the nearest QTL peak and the nearest TRDL peak using Spearman rank correlation tests. Genetic differentiation was further tested for associations with local recombination rate, measured as the genetic map distance to the nearest mapped marker, and with genetic diversity within species, measured as each of UHe, Ar and MAF using Spearman rank correlation tests. Marker loci on linkage groups without QTLs were assigned large QTL distance values of 50 cM in order to include them as part of these association tests. 

## Results

### Quantitative trait locus mapping and analysis

 The two parent species, *S. aethnensis* and *S. chrysanthemifolius* , differed significantly for 22 of the 25 traits. The exceptions were flowering time, leaf number and selfed seed-set (Traits 1, 3 and 18) ** [see Supporting Information—Table S2 and Fig. S1 ] ** . We surmise that the lack of flowering time difference in the glasshouse compared with field observations reflects the importance of environmental conditions for the expression of this trait. For example, suitable growing conditions at the onset of spring start later in higher elevation *S. aethnensis* habitat than lower elevation *S. chrysanthemifolius* habitat. In summary, *S. aethnensis* differed from *S. chrysanthemifolius* in being shorter and less branched, possessing smaller, less dissected leaves (i.e. having entire or slightly lobed edges), and fewer but larger capitula that produced larger seed. Significant differences between the mean of the F _2_ AC family and those of one or both parent species were also evident for all traits apart from pollen viability and selfed seed-set (Traits 16 and 18) ** [see Supporting Information—Table S2 ] ** . The means of the F _2_ AC family for all traits were neither significantly higher nor lower than the means of both parents. Paired-trait correlations are summarized in **Supporting Information—Table S3** . Overall, 4.3, 2.7 and 11 % of pairs of traits were significantly correlated after correction for multiple testing among wild sampled *S. aethnensis* , wild sampled *S. chrysanthemifolius* and the F _2_ AC mapping family, respectively. Instances of non-independence between traits were reduced by dropping highly correlated traits and traits used to calculate compound characters, leaving a subset of 13 independent traits for QTL analysis. 

**Figure 1.**
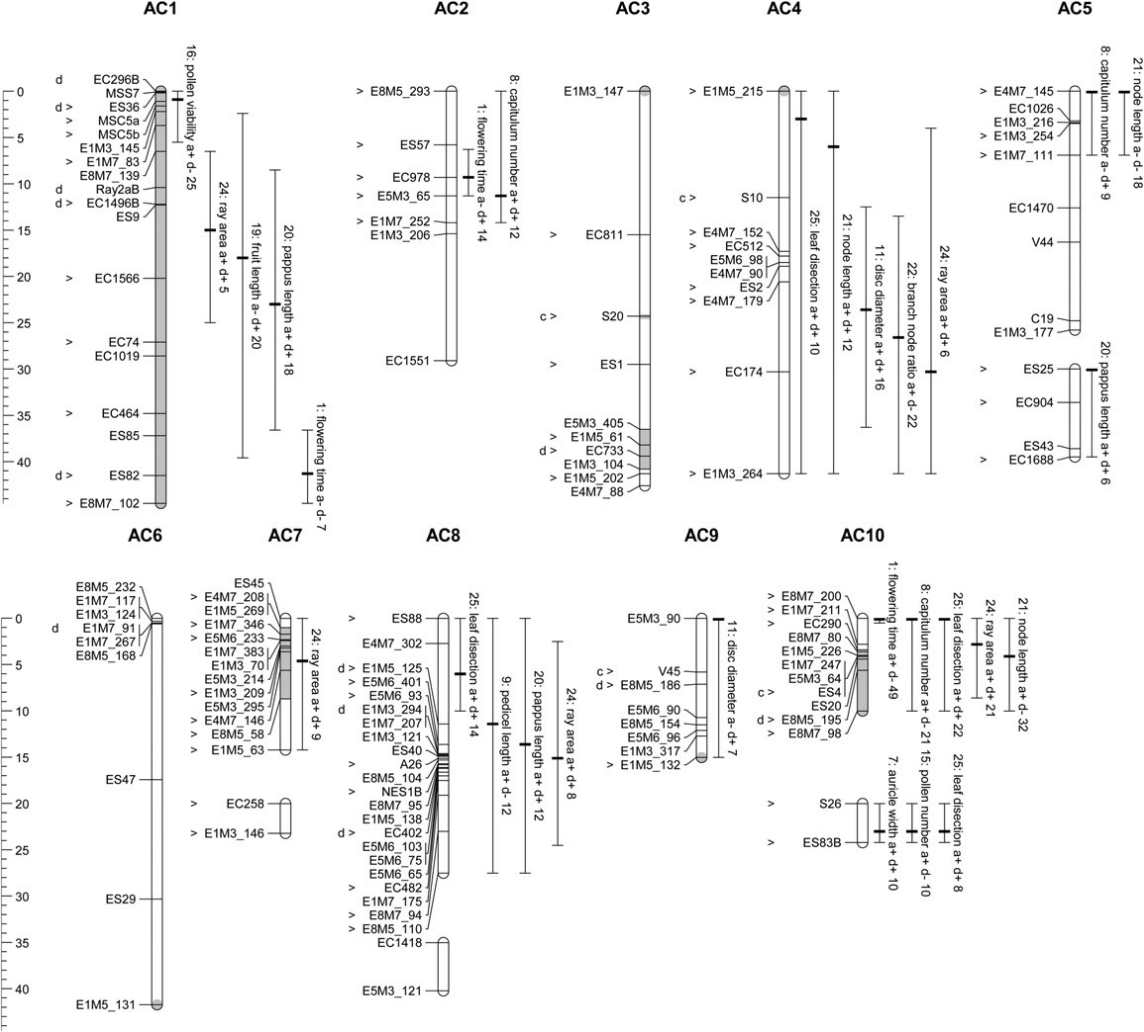
Genetic map of a reciprocal F _2_ *S. aethnensis* and *S. chrysanthemifolius* mapping family showing quantitative trait loci identified by MIM and marker loci that were significantly divergent or convergent between species. Map distances in Kosambi centiMorgans are shown in the scale bar to the left of linkage groups. Linkage groups are represented by vertical bars with mapped locus positions indicated with horizontal lines. Weakly linked linkage groups (<4 LOD or >20 cM) that probably belong to the same chromosome are aligned vertically. Grey shading on linkage groups indicates regions exhibiting significant TRDLs. Locus names are listed to the left of linkage groups and mapped QTLs are listed to the right. ‘c’ or ‘d’ listed to the left of locus names indicates if locus was identified to be significantly convergent or divergent based on genetic differentiation analysis across sample populations, while the greater than symbol to the left of locus names indicates if the locus was included in QTL analysis. Quantitative trait loci were identified by MIM with significance testing by BIC model comparisons. Quantitative trait loci 2-LOD ranges are indicated with vertical lines with a bold horizontal line indicating the highest LOD score position. Quantitative trait locus summary information includes trait names, ‘a’ or ‘d’ each followed by ‘+’ or ‘−’ indicating additive or dominance effects and their direction of effect supporting or opposing the observed species difference, respectively, and the PVE.

 Significant QTLs for each trait were detected and characterized by LOD score, map position, two LOD confidence intervals, size of additive, dominance and epistatic effects, and percentage variance explained (PVE). A total of 29 significant QTLs were detected across the 13 traits examined with mean QTL effect size of 15 % (Table  1 , Fig.  1 ). Quantitative trait loci were distributed across all major linkage groups except AC3 and AC6, with one to five QTLs detected for each trait (Fig.  1 ). The mean PVE of all identified QTLs per trait was 33.5 % (range = 10.0–69.8 %). 

**Table 1.** Summary QTL results from a MIM analysis of a reciprocal F _2_ *S. aethnensis* and *S. chrysanthemifolius* mapping family. Quantitative trait locus LG and position are the linkage group and maximum likelihood of odds score (LOD) cM position of significant QTLs identified from MIM. Quantitative trait locus cM interval is the 2-LOD cM interval around the maximum LOD value that is indicated in parentheses. The additive and dominance effects are in the same units as trait measures. Positive additive effects support the direction of the species difference and vice versa for negative effects, while positive dominance effects indicate that *S. aethnensis* alleles are dominant and vice versa for negative effects. The PVE is shown in parentheses. Epistatic interactions show the loci for each trait (numbered in the order they appear in the table) with significant interactions with the additional PVE shown in parentheses.

Trait ID number: trait (units)	QTL LG and position (cM)	QTL cM interval (max LOD)	Additive effect (PVE)	Dominant effect (PVE)	Total PVE	Epistatic interactions (PVE)
1: Time from first true leaf to flowering (days)	AC1; 41.3	36.6–44.5 (6.19)	−3.41 (5.7)	−2.31 (1.3)	7	2 × 3 (5.4)
	AC2; 9.3	6.3–11.3 (9.22)	−5.27 (14.3)	0.13 (0)	14.3	
	AC10A; 0	0–0.5 (15.35)	9.02 (46.2)	−3.56 (2.3)	48.5	
7: Primary stem midleaf auricle width (mm)	AC10B; 3	0–4.2 (2.21)	0.52 (7.3)	0.41 (2.7)	10	
8: Primary inflorescence capitulum number (count)	AC2; 11.3	0–14.2 (2.5)	1.29 (8.5)	0.96 (3.6)	12.1	
	AC5A; 0	0–6.9 (2.57)	−1.34 (9)	0.36 (0.4)	9.4	
	AC10A; 0	0–10 (4.89)	2.14 (20.7)	−0.43 (−0.1)	20.6	
9: Primary capitulum pedicel length (cm)	AC8A; 11.4	0–27.5 (2.5)	0.26 (11.7)	−0.07 (0.4)	12.1	
11: Primary capitulum disc diameter (mm)	AC4; 23.6	12.5–36.3 (3.52)	0.05 (6.4)	0.08 (9.8)	16.2	
	AC9; 0	0–15 (2.13)	−0.05 (6.2)	0.03 (1.2)	7.4	
15: Mean pollen number (per 3/40 florets)	AC10B; 3	0–4.2 (2.03)	7.79 (6.2)	−8.94 (3.8)	10	
16: Mean pollen viability (proportion)	AC1; 0.9	0–5.5 (5.4)	0.01 (−1.2)	−0.19 (25.9)	24.7	
19: Mean fruit length (mm)	AC1; 18	2.4–39.6 (2.89)	−0.21 (−5.8)	0.36 (26.1)	20.3	
20: Mean pappus length (mm)	AC1; 23	8.5–36.6 (4.6)	0.15 (5.9)	0.32 (12)	17.9	
	AC5B; 0	0–9.5 (2.09)	0.2 (5.6)	0.01 (0)	5.6	
	AC8A; 13.6	0–27.5 (3.43)	0.27 (10.6)	0.12 (1.3)	11.9	
21: Primary stem node length, height to leaf number ratio (cm)	AC4; 6	0–41.3 (2.8)	0.09 (8.6)	0.1 (2.9)	11.5	
	AC5A; 0	0–6.9 (6.37)	−0.12 (18)	−0.01 (0.1)	18.1	
	AC10A; 4.1	0–10 (11.57)	0.16 (30.1)	−0.11 (2)	32.1	
22: Branch number to leaf number (proportion)	AC4; 26.6	13.5–41.3 (4.3)	0.1 (9.9)	−0.17 (12.1)	22	
24: Primary capitulum ray display area (mm ^2^ )	AC1; 15	6.5–25 (6.75)	4.69 (3.3)	3.37 (1.9)	5.2	1 × 3 (5.1)
	AC4; 30.3	4–41.3 (2.07)	6.9 (6.2)	0.25 (0.1)	6.3	1 × 4 (7.8)
	AC7A; 4.6	0–14.2 (6.45)	5.79 (2.1)	16.83 (7.1)	9.2	
	AC8A; 15.1	2.5–24.5 (5.5)	8.13 (8)	5.67 (−0.2)	7.8	
	AC10A; 2.8	0–8.6 (8.01)	15.43 (20.9)	0.54 (0.2)	21.1	
25: Primary stem midleaf dissection, perimeter to area ratio (per mm)	AC4; 3	0–41.3 (2.02)	0.04 (6.9)	0.04 (2.9)	9.8	2 × 3 (10.1)
	AC8A; 6	0–10 (7.29)	0.04 (7.7)	0.05 (6.3)	14	
	AC10A; 0	0–10 (9.01)	0.06 (21.1)	0.01 (0.5)	21.6	
	AC10B; 3	0–4.2 (2.6)	0.02 (3.9)	0.04 (4.4)	8.3	

 Four pairs of QTLs exhibited significant epistatic interaction effects with a mean PVE of 7.1 % (range = 5.1–10.1; Table  1 ). The MtCIM analysis of all traits identified three significant and three almost significant (within 1 LOD of the permutation threshold of 14.82 LOD) pleiotropic loci with multiple trait effects (Table  2 ) ** [see Supporting Information—Table S5 ] ** . These potential pleiotropic loci overlapped with the 2-LOD intervals of 14/29 of the individual trait QTLs, with up to four traits affected at each site (Table  1 ). Thus, 14 QTLs for eight traits exhibited pleiotropic effects. The ‘sampling without replacement’ method using the 16.5 cM interval size found four trait pairs, auricle width and pollen number, capitulum number and node length, capitulum number and flowering time, and node length and leaf dissection, that showed significantly coincident QTL locations ** [see Supporting Information—Table S6 ] ** . Sampling without replacement analyses using a range of shorter interval sizes found similar evidence for coincident QTL locations, but failed to find any previously identified TRDLs that were significantly coincident with trait QTLs ** [see Supporting Information—Table S6 ] ** . 

**Table 2.** Summary QTL results from a MtCIM analysis of a reciprocal F _2_ *S. aethnensis* and *S. chrysanthemifolius* mapping family. Locus LG and peak cM are the linkage group and maximum likelihood odds score (LOD) cM position of a locus that affects the expression of multiple traits. Locus 2-LOD interval is the 2-LOD interval around the peak for the locus with the maximum LOD value indicated in parentheses. Overlapping quantitative trait loci from the MIM analysis are shown for comparison [see Table  1 and **Supporting Information—Table S4** for more details about QTLs].

Locus LG, peak cM position	Locus 2-LOD interval (peak LOD)	Overlapping QTL LOD intervals
AC1, 0.9	0–6.5 (14.52)	16: Mean poor pollen
AC1, 29.9	26.9–32.9 (14.35)	19: Mean fruit length 20: Mean pappus length
AC2, 5.8	1.5–11.3 (14.1)	1: Time from first true leaf to flowering 8: Primary inflorescence capitulum number
AC4, 3	1.8–4.2 (17.69)	21: Primary stem node length 24: Primary capitulum ray display area 25: Primary stem midleaf dissection
AC5A, 3	0–6.9 (17.24)	8: Primary inflorescence capitulum number 21: Primary stem node length
AC10A, 8.6	3.4–10 (28.6)	8: Primary inflorescence capitulum number 21: Primary stem node length 24: Primary capitulum ray display area 25: Primary stem midleaf dissection

### Genetic diversity analysis

 Both species exhibited similar levels of genetic diversity, with the highest diversity recorded for anonymous SSRs, followed in turn by EST-SSRs, EST-indels and AFLPs, and other dominant markers ** [see Supporting Information—Tables S7 and S8 ] ** . Overall, inbreeding coefficients were not significantly different from zero in either species indicative of random mating ( *F*_IS_ = 0.02 and 0.06 in *S. aethnensis* and *S. chrysanthemifolius* , respectively) ** [see Supporting Information—Table S7 ] ** . The two species were significantly genetically differentiated across all marker types with overall *F*_ST_ of 0.28 and 0.31 observed for dominant markers and codominant markers, respectively ** [see Supporting Information—Tables S7 and S8 ] ** . 

**Figure 2.**
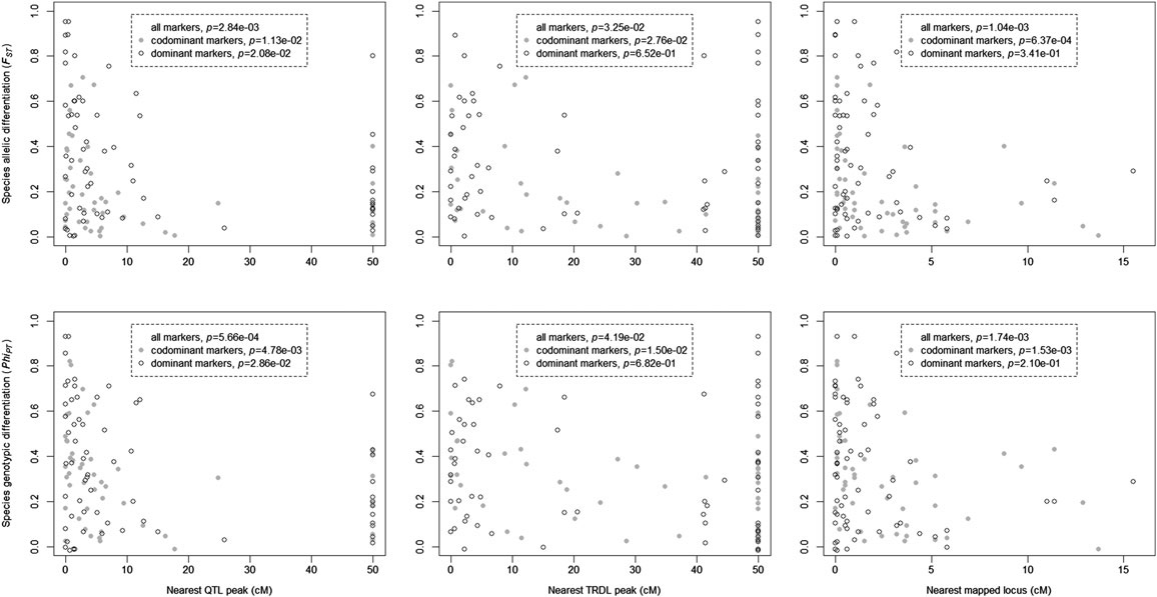
Relationships between genetic differentiation and genetic map distance to the nearest QTL peak, the nearest TRDL or the nearest mapped marker. Presented *P* values summarize Spearman rank correlation tests. All significant associations were negative. Sample sizes were 48 codominant loci and 63 dominant loci. Loci on linkage groups without a QTL or TRDL peak were assigned an unlinked genetic map distance of 50 cM.

**Figure 3.**
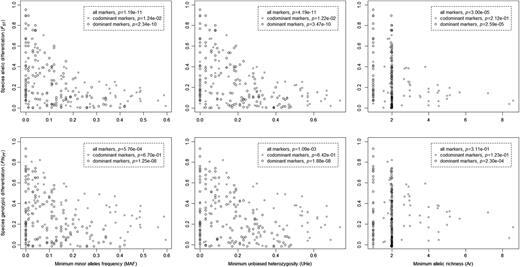
Relationships between genetic differentiation and genetic diversity of wild sampled *S. aethnensis* and *S. chrysanthemifolius* . Presented *P* values summarize Spearman rank correlation tests. All significant associations were negative. Sample sizes were 65 codominant loci and 145 dominant loci.

 Bayesian analyses of species differentiation showed that 4.7 % of codominant markers, but 0 % of dominant markers, were divergent outliers and that the same percentages of each marker type were significantly convergent outliers (Table  3 ). When population information was included in these analyses, the tests were more sensitive and identified 7.8 and 5.3 % of significantly divergent codominant and dominant markers, respectively, and 4.7 and 0.8 % of significantly convergent codominant and dominant markers, respectively (Fig.  1 , Table  3 ) ** [see Supporting Information—Table S9 ] ** . Significant outlier loci were distributed across most linkage groups of the genetic map (Fig.  1 ) and showed no evidence of clustering according to a one-way binomial test of an excess of neighbouring pairs of outlier markers ( *P* = 0.1754). However, significant negative associations between measures of species differentiation for marker loci and the genetic map distance from the nearest QTL peak were present (Fig.  2 ). Similarly, there was evidence for negative associations between marker gene differentiation and genetic map distance from the nearest TRDL (Fig.  2 ). A significant negative association between genetic differentiation and low recombination in the form of genetic map distance to closest neighbouring mapped locus was also found (Fig.  2 ). Also, significant negative associations were present between genetic differentiation between species and the various intraspecific genetic diversity measures (Fig.  3 ). In general, all of these associations were stronger for codominant than for dominant markers. 

**Table 3.** Summary of numbers of marker loci identified as significantly divergent or convergent outliers between *S. aethnensis* and *S. chrysanthemifolius* . Samples tested included all samples scored according to species (Species), populations (Populations) or data subsets of only *S. aethnensis* populations or only *S. chrysanthemifolius* populations. Only polymorphic loci with minor allele frequencies >0.05 were included in analyses. In the case of dominant loci, allele frequency was calculated assuming within-population Hardy–Weinberg equilibrium. Loci were considered significantly divergent or convergent with log _10_ Bayes Factor statistics >1.

Samples tested	No. codominant loci tested	No. dominant loci tested	No. codominant loci divergent (%)	No. codominant loci convergent (%)	No. dominant loci divergent (%)	No. dominant loci convergent (%)
Species	64	132	3 (4.7)	3 (4.7)	0 (0.0)	0 (0.0)
Populations	64	132	5 (7.8)	3 (4.7)	7 (5.3)	1 (0.8)
*S. aethnensis*	53	115	0 (0.0)	0 (0.0)	0 (0.0)	0 (0.0)
*S. chrysanth emifolius*	61	110	1 (1.6)	0 (0.0)	0 (0.0)	0 (0.0)

## Discussion

### Quantitative trait locus architecture

 Quantitative trait locus analysis identified 1–5 QTLs per trait and 29 QTLs in total for the 13 independent traits examined that distinguish the two *Senecio* species. In addition to resolving the primary effects of individual QTLs, MIM and MtCIM analyses provided evidence for epistatic interactions between four pairs of QTLs and possible pleiotropic effects at six loci affecting eight traits (Tables  1 and 2 ). Sampling without replacement tests indicated that QTL map locations were significantly clustered across the genetic map, with significant physical associations evident for four trait pairs ** [see Supporting Information—Table S5 ] ** .  Chapman *et al.* (2016)  reported similar clustering of QTLs for species differences in an independent mapping study of *S. aethnensis* and *S. chrysanthemifolius* . However, their study did not investigate patterns of epistasis and pleiotropy. Regardless of whether the observed interactions between QTLs are due to epistasis or pleiotropy or physical linkage, they indicate that different traits are not genetically independent and that divergent selection acting on one trait would therefore also affect other traits. 

 A QTL architecture involving extensive physical and epistatic interactions between QTLs, together with pleiotropic effects of individual QTLs, should limit introgression between the two *Senecio* species on Mount Etna since hybridization would tend to break up gene complexes that control the expression of adaptive phenotypes in each species (  Fenster *et al.* 1997  ;  Turelli *et al.* 2001  ). The complex genomic architecture of interspecific divergence revealed in *Senecio* might reflect the evolutionary outcome of selection for non-independence of different QTLs controlling traits under divergent selection ( Kirkpatrick and Barton 2006 ;  Nosil *et al.* 2009  ; Nosil and Feder 2012 ; Yeaman 2013 ). Limiting recombination seems to be the crucial factor permitting interacting QTLs to evolve into divergent co-adapted QTL complexes in the presence of gene flow. This can be achieved either through chromosomal rearrangement that causes recombination between rearranged regions to become deleterious (  Feder *et al.* 2003  ; Lowry and Willis 2010 ; Twyford and Friedman 2015 ) or by evolution towards increased physical proximity (coincidence) through locally biased persistence, establishment or translocation of QTLs ( Via and West 2008 ;  Nosil *et al.* 2009  ; Yeaman 2013 ). Genetic mapping indicates that *S. aethnensis* and *S. chrysanthemifolius* are not distinguished by major genome rearrangements (  Brennan *et al.* 2014  ), which instead emphasizes the importance of QTL coincidence for this system (our results and those of  Chapman *et al.* 2016  ). 

 While TRDLs were not significantly coincident with QTLs for any trait according to the ‘sampling without replacement’ method, a QTL affecting pollen viability co-located with a TRDL of large effect in linkage group AC1 (Fig.  1 ). This finding is of interest as it adds to the result previously reported by  Chapman *et al* . (2016)  of co-localization of TRDLs with QTLs affecting F _2_ hybrid necrosis. Hybrid incompatibilities, such as decreased F _2_ pollen viability and hybrid necrosis and their associated TRDLs, are expected to limit introgression across large genomic regions allowing further divergence of these regions during speciation ( Barton and Bengtsson 1986 ; Barton and de Cara 2009 ). 

### Non-random patterns of divergence across the genome

 Levels of molecular genetic diversity were similar in wild samples of both *Senecio* species, while genetic differentiation between species was moderate. Genetic diversity decreased from estimates based on anonymous SSRs to EST-SSRs, to EST-indels to AFLPs, corresponding to the expected ability of each marker type to resolve allelic variation ** [see Supporting Information—Tables S6 and S7 ] ** . Low levels of genetic differentiation between the two species were also reported by  Muir *et al.* (2013)  ,  Osborne *et al.* (2013)  and  Chapman *et al.* (2013)  , based on surveys of microsatellite and sequence variation. We identified a small percentage of loci that were either significantly divergent or convergent (up to 7.8 %) between species, dependent on the marker set analysed (Table  3 ) ** [see Supporting Information—Table S9 ] ** . This value is slightly greater than the 2.25 % of outliers from a study of 8854 loci recorded by  Chapman *et al.* (2013)  based on a comparison of the two species’ transcriptomes, but the two findings are probably within the bounds of error given the different numbers of loci examined. More discussion about the functions of significantly divergent or convergent loci is provided in the Supplementary information . Inevitably, the 196 marker loci for which patterns of differentiation were compared to detect significant divergence between species in the present study provide only a very coarse-grained perspective across the whole genome, and many of the true genetic targets of selection will not have been surveyed. 

 Reduced effective gene flow in the vicinity of selected loci is often used to explain significantly differentiated loci and ‘islands of divergence’ ( Wu 2001 ; Feder and Nosil 2010 ). In support of this hypothesis, significant associations were found between interspecific genetic differentiation and genetic map distance to QTLs and TRDLs (Fig.  2 ). These associations were negative with more highly differentiated loci positioned closer to QTLs or TRDLs. These results support previous findings that selection against hybridization is important for maintaining species distinctiveness across the *Senecio* hybrid zone on Mount Etna (  Brennan *et al.* 2009  ;  Chapman *et al.* 2013  , 2016 ). However, independently of gene flow, within-species directional selection can also generate the same pattern of divergence via species-specific reductions in diversity ( Cruikshank and Hahn 2014 ). The latter is amplified when it occurs in regions of low recombination as it causes longer genomic regions to be affected by selection at linked markers. In accordance with these hypotheses and in agreement with the findings of  Chapman *et al.* (2016)  , we also found evidence for intraspecific selection in the form of significant negative associations between interspecific differentiation and local recombination, and between interspecific differentiation and intraspecific genetic diversity (Figs  2 and 3 ). It is plausible that *S. aethnensis* and *S. chrysanthemifolius* experience distinct localized selection pressures related to the very different environments they occupy at different elevations on Mount Etna. Such within-species selection would be expected to reduce within-species genetic diversity in the genomic regions experiencing selection. These findings, therefore, suggest a role for environment-specific extrinsic selection in maintaining the cline with elevation on Mount Etna. While this pattern of diversity might also signal past periods of isolation facilitating divergence, other genetic studies suggest that gene flow between the two species has probably been continuous throughout their history (  Chapman *et al.* 2013  ;  Osborne *et al.* 2013  ;  Filatov *et al.* 2016  ). 

## Conclusions

 Our study shows that phenotypic divergence across the elevational gradient on Mount Etna involves divergence of multiple quantitative traits controlled by numerous interacting genes (QTLs). A breakdown in the complex genetic architecture of these traits following hybridization would be expected to reduce the fitness of most hybrid offspring and therefore contribute to introgression barriers between the two *Senecio* species. Our combined analyses of genetic differentiation, QTLs and TRDLs emphasize that divergence is non-randomly distributed across the genomes of these species and that both selection against hybrids between species and locally maladapted individuals within species will act to maintain phenotypic divergence between the two species in the face of gene flow. 

## Sources of Funding

The research was funded by a Natural Environment Research Council (NERC) Grant NE/D014166/1 to R.J.A. as principal investigator. A.C.B. was supported during part of the writing of this paper by funding from European Commission 7th Framework Programme Capacities Work Programme (FP7-REGPOT 2010-1), Grant No. 264125 EcoGenes.

## Contributions by the Authors

R.J.A., A.C.B. and S.J.H. designed the research. A.C.B. performed the experiments and analysis. A.C.B. wrote the first draft and A.C.B., R.J.A. and S.J.H. contributed to revisions.

## Conflict of Interest Statement

None declared.

## Supporting Information

The following additional information is available in the online version of this article —

**Table S1.** Information on wild sampled populations of *S. aethnensis* and *S. chrysanthemifolius* . 

**Table S2.** Summary quantitative trait results for *S. aethnensis* , *S. chrysanthemifolius* and a reciprocal F _2_ *S. aethnensis* and *S. chrysanthemifolius* mapping family. 

**Table S3.** Paired-trait correlations in (a) F _2_ AC progeny, (b) *Senecio aethnensis* , (c) *S. chrysanthemifolius* and (d) all three samples. 

**Table S4.** Comparison of summary QTL results for a CIM and MIM analysis of a reciprocal F _2_ *S. aethnensis* and *S. chrysanthemifolius* mapping family. 

**Table S5.** Summary QTLs results from a MtCIM analysis compared with single trait QTL analyses of a reciprocal F _2_ *S. aethnensis* and *S. chrysanthemifolius* mapping family. 

**Table S6.** (a) ‘Sampling without replacement’ test results for paired-trait QTL coincidence, (b) permutation tests of overall paired-trait QTL coincidence using different QTL and TRDL datasets and genetic map interval sizes. 

**Table S7.** Summary population genetic statistics for AFLPs and other dominantly scored molecular genetic markers from *S. aethnensis* and *S. chrysanthemifolius* samples. 

**Table S8.** Summary population genetic statistics for codominantly scored molecular genetic markers from *S. aethnensis* and *S. chrysanthemifolius* samples. 

**Table S9.** Expressed sequence tag loci showing evidence for divergent or convergent selection between *S. aethnensis* and *S. chrysanthemifolius* . 

**Figure S1.** Boxplots summarizing quantitative trait results for *S. aethnensis* , *S. chrysanthemifolius* and a reciprocal F _2_ mapping family that were included in the QTL analysis. Trait numbers in titles correspond to the trait numbering system of Table  1 . Bold horizontal lines indicate median values. Boxes indicate 25–75 percentile range. Lines indicate the range of values within 1.5 times the upper and lower quartiles, respectively. Points indicate values more extreme than 1.5 times the upper and lower quartiles. Asterisks indicate the trait values of the mapping family parents. No mapping family parental values were available for flowering time as these individuals were vegetatively propagated for comparison with their progeny. 

**Figure S2.** Genetic map of a reciprocal F _2_ *S. aethnensis* and *S. chrysanthemifolius* mapping family showing quantitative trait loci identified by CIM and marker loci that were significantly divergent or convergent between species. Map distances in Kosambi centiMorgans are shown in the scale bar to the left of linkage groups. Linkage groups are represented by vertical bars with mapped locus positions indicated with horizontal lines. Weakly linked linkage groups (<4 LOD or >20 cM) that probably belong to the same chromosome are aligned vertically. Grey shading on linkage groups indicates regions exhibiting significant TRDLs. Locus names are listed to the left of linkage groups and mapped QTLs are listed to the right. ‘c’ or ‘d’ listed to the left of locus names indicates if that locus was identified as significantly convergent or divergent based on genetic differentiation analysis across sample populations, while greater than symbol to the left of locus names indicates if the locus was included in QTL analysis. Quantitative trait loci were identified by CIM with significance determined if the LOD score exceeded the 0.95 quantile of 1000 data permutations. Quantitative trait loci 2-LOD interval ranges are indicated with vertical lines with a bold horizontal line indicating the highest LOD score position. Quantitative trait locus summary information includes trait names, ‘a’ or ‘d’ each followed by ‘+’ or ‘−’ indicating additive or dominance effects and their direction of effect supporting or opposing the observed species difference, respectively, and the PVE. 

**Supplementary information.** Additional text describing the genetic map, TRD analysis, composite interval mapping, QTL sign tests, genetic diversity analyses and discussion of the functions of significantly divergent and convergent loci. 

## Supplementary Material

1504_0_supp_1_nsl1yf

1504_0_supp_12_nsnxrl

1504_1_supp_1_nzrs2d

1504_1_supp_1_nzrs7h
