# Supplementary figures and images for: Genomic architecture of phenotypic divergence between two hybridizing plant species along an elevational gradient
Source: AoB Plants. 2015 Aug 18;8:plw022. doi: 10.1093/aobpla/plw022 (PMC4887755; doi:10.1093/aobpla/plw022)

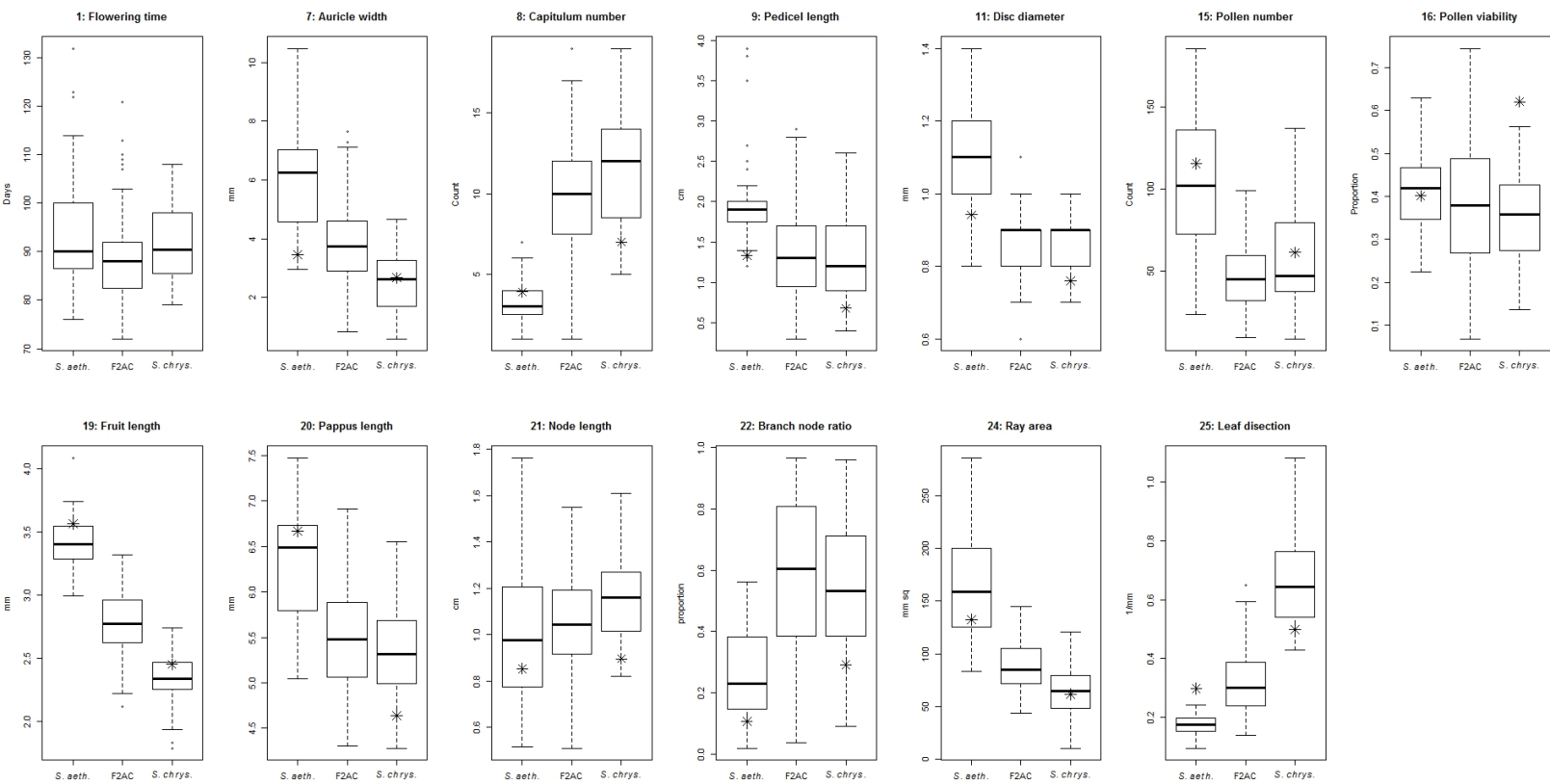

Supplement: 1504_0_supp_1_nsl1yf [file 1504_0_supp_1_nsl1yf.pdf]

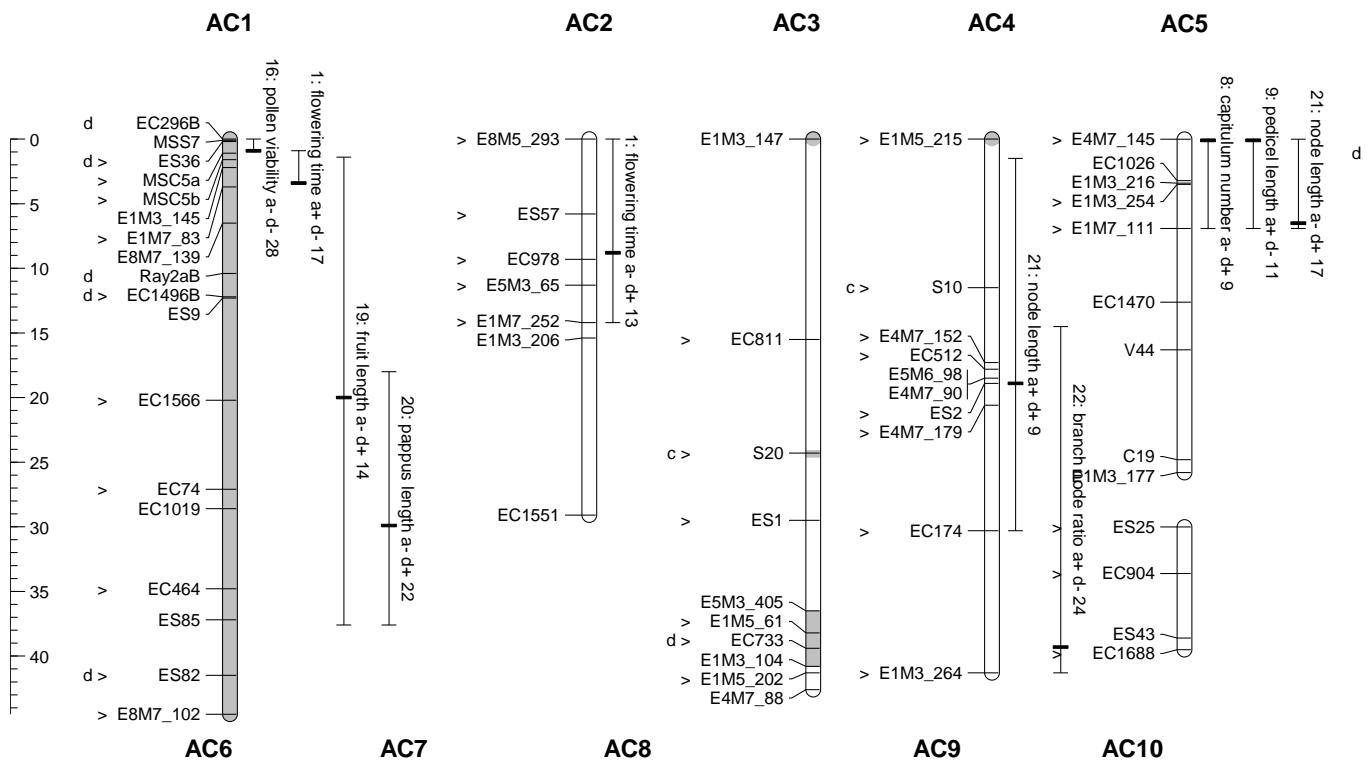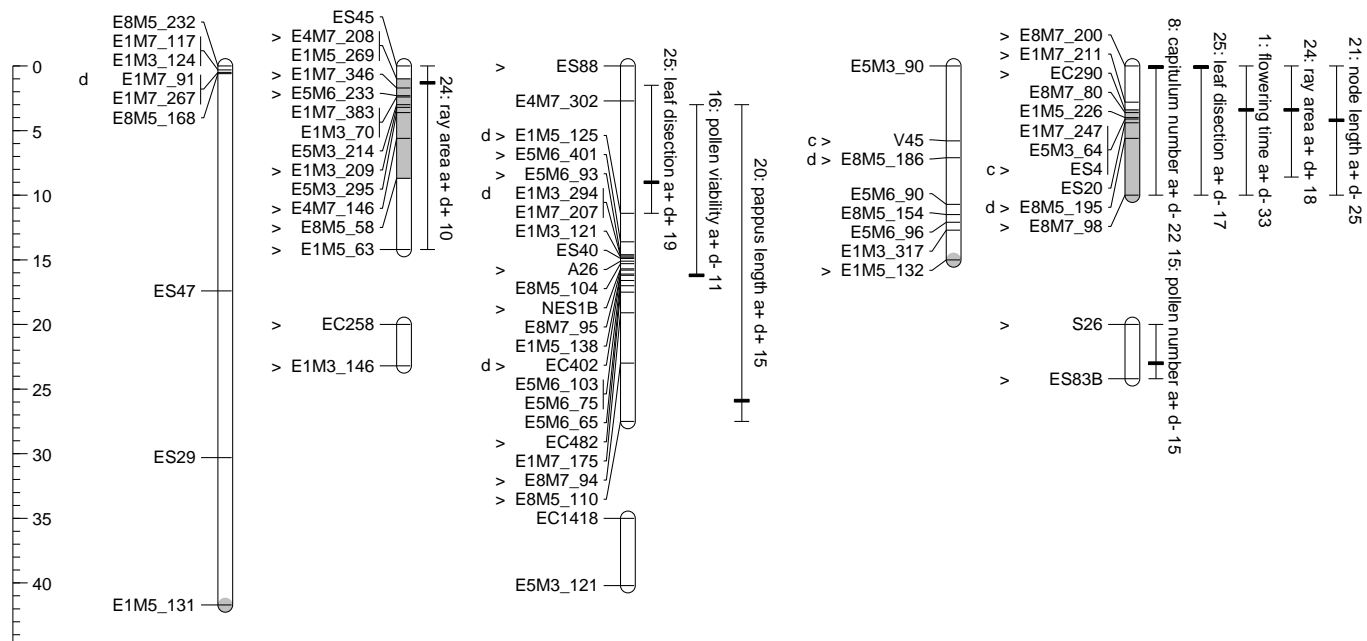

Supplement: 1504_0_supp_12_nsnxrl [file 1504_0_supp_12_nsnxrl.pdf]
