# Supplementary material for: Genomic architecture of phenotypic divergence between two hybridizing plant species along an elevational gradient
Source: AoB Plants. 2015 Aug 18;8:plw022. doi: 10.1093/aobpla/plw022 (PMC4887755; doi:10.1093/aobpla/plw022)
Supplement: 1504_1_supp_1_nzrs2d [file 1504_1_supp_1_nzrs2d.pdf]

Genomic architecture of phenotypic divergence between two hybridizing plant species along an elevational gradient: Supplementary Information.

Genetic mapping

A genetic linkage map was constructed from the segregation of alleles at marker loci in the F_2AC family using the demonstration version of Joinmap v4.0 (Van Ooijen 2001). In the analysis, the F_2AC was treated as an outcrossed mapping family (CP type). Linkage groups were identified at greater than four logarithm of odds (LOD) score with less than 20 Kosambi centiMorgan (cM) map distance units between loci. The most likely linkage phase of heterozygous F_0 parental alleles was assigned to each of the two parental chromosomes for each group of linked loci. Default regression mapping algorithm parameters were used to determine the optimum orders and distances of markers on linkage groups. Map quality was assessed by examining goodness of fit G2 statistics and markers responsible for incompatible linkage interactions were removed to generate linkage groups with high map support. Diagrams of linkage groups were constructed using MapChart v2.2 (Voorrips, 2002).

Map length was estimated by adding twice the mean marker distance to the length of each linkage group to account for ends beyond the terminal markers (Fishman et al., 2001) and also by multiplying the length of each linkage group by the correction factor (marker number +1)/(marker number -1) (Chakravarti et al., 1991). Map coverage in terms of the percentage of the genome that is within 5 or 10 cM of a mapped marker was assessed according to the formula: 1-exponent((-2*distance*marker number)/map length)) (Fishman et al., 2001).

Transmission ratio distortion

Genotype frequencies at each mapped marker locus in the entire F_2AC mapping family were tested for Mendelian segregation using chi square tests with Microsoft Excel 2003 (Microsoft corp, 2003). The distribution of loci showing transmission ratio distortion (TRD) was examined by plotting per locus chi square test results onto their genetic map positions. The number of distinct TRD loci (TRDLs) across the genetic map was assessed as the number of clusters of markers at a map distance of less than 10 cM from each other showing distorted genotype segregation at a single-locus 95% confidence level. The most likely map position of a TRDL was interpreted as the map position of the locus exhibiting the greatest TRD within each cluster of distorted markers. Some markers showing TRD were isolated by more than 10 cM from the nearest marker also exhibiting TRD. These markers were considered as possibly representing different TRDLs, albeit with weaker supporting evidence. Transmission ratio distortion affected ~27% of mapped markers that were clustered into four transmission ratio distortion loci (TRDLs) located on linkage groups: AC1, AC3 (distal position), AC7A, and AC10A, as well as an additional five putative TRDLs made up of isolated segregation distortion markers on linkage groups: AC3 (proximal and central widely separated locations), AC4, AC6, and AC9 (Brennan *et al.* 2014).

Composite Interval Mapping (CIM)

For comparison with MIM, composite interval mapping (CIM; Zeng 1994), a widely used QTL mapping method, was performed using a scanning interval of 3 cM and default automatic selection by forward regression of five cofactor loci outside an interval window of 10 cM. Significance of QTL LOD scores was tested with 1000 permutations of trait values (Churchill and Doerge 1994). CIM also controls for possible marker-trait associations in the rest of the gentic map outside the focus

region through the use of cofactors, but unlike MIM, CIM does not account for the presence of or interactions with other identified QTLs. A total of 29 and 20 significant QTLs were detected across the 13 traits examined using MIM and CIM analyses, respectively (Table 1, Figure 1, Table S4, Figure S2). Eighteen of the same QTLs were identified by both analyses. The larger number of QTLs resolved by MIM was due to the greater sensitivity of this form of analysis to detect significant QTLs with smaller effect sizes (15% versus 17% mean QTL size of effect in MIM and CIM, respectively). Because of the increased detection sensitivity of MIM, the main text was focused on the QTL results obtained with MIM, noting that results obtained with CIM were qualitatively similar.

QTL sign tests

Sign tests for an excess of QTLs showing directions of effect that support the overall observed phenotypic divergence are a simple but powerful test for divergent selection versus neutral divergence (Orr 1998; Lexer *et al.* 2005). In addition, dominance tests for bias of dominant QTL alleles from one cross direction can provide information on the species that has responded most to divergent selection according to Haldane's sieve hypothesis that new dominant alleles are more visible to selection (Orr and Betancourt 2001; Pannell *et al.* 2005).

A QTL sign test of the extent of the bias of QTLs supporting the observed trait difference between species was performed using R (one tailed binomial exact test) to test the hypothesis of an excess of large positive effect QTL effects under directional selection compared to neutral divergence (QTLST; Orr 1998). In order to have a sufficient number of QTLs to apply the test with reasonable power, all traits for which multiple QTLs were detected were used to detect an overall signal of divergent selection (Rieseberg *et al.* 2002; Lexer *et al.* 2005). The QTL effect sizes

were standardized across different traits by dividing by the F_0 parental difference. The sum of standardized effect sizes was used as the test statistic against which to compare the distribution of 10,000 random permutations of these effect sizes with equal probabilities for direction of effect. An equivalent QTL test of bias in direction of dominance of QTL alleles between both species was also performed to test the hypothesis of an excess of large dominant effect QTL alleles when divergent selection is asymmetrical; *i.e.* one species in the pair has experienced greater evolutionary change (Pannell *et al.* 2005).

While the direction of effect of most QTLs supported the observed trait difference between *S. aethnensis* and *S. chrysanthemifolius* (23 out of 29 QTLs), a QTL sign test of QTL additive effect sizes for traits with multiple QTLs did not find significant evidence for divergent selection between the species (p = 0.1739). Similarly, while most QTLs showed dominant *S. aethnensis* alleles (20 out of 29), the QTL sign test of QTL dominance effect sizes for traits with multiple QTLs did not find significant evidence for a bias in QTL dominance expression between species (p = 0.3575). Future studies with larger mapping families allowing more smaller effect size QTLs to be identified would provide QTL sign tests with more power to test if divergent selection is contributing to species distinctiveness (Anderson and Slatkin 2003).

Genetic diversity analysis

Following DNA extraction according to Brennan et al. (2009), plants were genotyped across 127 marker loci comprising 77 AFLPs, eight SSRs, and 42 EST-SSRs and indel molecular markers as described by Brennan et al. (2014). Two of the indel markers were developed from published polymorphic Senecio gene sequences. These were SSP, which encodes a stigma-specific peroxidase involved in pollination (McInnis et al. 2005) and Ray2a, which encodes a cycloidea-like transcription factor

involved in the control of ray floret development in Senecio (Kim et al. 2008). If molecular markers are developed from genes of known function, e.g. simple sequence repeats (microsatellites) developed from expressed sequence tags (EST-SSRs), they can potentially provide information on the functions of genomic differences between hybridizing species and the candidate genes involved (Bouck and Vision 2007; Stinchcombe and Hoekstra 2008; Galindo *et al.* 2010).

Patterns of differentiation across loci were investigated to detect both strongly and weakly differentiated outlier loci, which could be considered candidates for divergent or convergent selection, respectively, using Bayescan (Foll and Gaggiotti 2008) as described in the main text. The EST and gene markers with associated sequence information that were found to be significantly divergent or convergent were BLAST searched against the NCBI nucleotide database (NCBI resource coordinators 2013; http://blast.ncbi.nlm.nih.gov/Blast.cgi) for all organisms and for the model plant, *Arabidospsis thaliana*, using the relaxed alignment tblastx option to identify orthologous genes and investigate their function.

Of the ESTs and genes of known function identified by the differentiation tests for selection, four (EC296B, ES36, EC1496B, and *Ray2a*) were located on linkage group AC1, a large genomic region affected by a strong TRDL acting against introgression of *S. aethnensis* alleles (Brennan *et al.* 2014). Marker locus EC296B showed the strongest transmission ratio distortion and is probably at or close to the TRDL itself. This EST is a member of the pathogenesis related plant defensin (PDF) gene family in *Arabidopsis thaliana* with probable expression in pollen according to the closest tBLASTx match in *Artemesia vulgaris* (Asteraceae). This suggests a role in plant defence and/or reproduction in *Senecio*. A possible functional role in pollen was also suggested by the location of EC296B within the 2-LOD confidence interval

of the pollen viability QTL (Fig. 1). Both putative functions of EC296B have previously been implicated in hybrid incompatibility in other species, with plant defence in particular considered as a potent source of Bateson-Dobzhansky-Muller (BDM) two-locus incompatibility interactions between diverging genomes (Moyle and Graham 2006; Bomblies and Weigel 2007; Bikard *et al.* 2009; Alcazar *et al.* 2010). Blast searches indicated that ES36 encodes a transcription factor while EC1496B probably encodes a tubulin component. Interestingly, EC1494B showed significantly greater expression in *S. chrysanthemifolius* relative to *S. aethnensis* in a previous transcriptome microarray study (Hegarty *et al.* 2009). However, reasons for the divergence of these ESTs remain unknown and neither of their functions suggests an obvious connection to hybrid incompatibility or divergent selection between the two *Senecio* species.

Ray2a is a *cycloidea*-like gene known to be involved in the control of ray floret ligule development in *Senecio* (Kim *et al.* 2008). Ray floret length and width are both significantly greater in *S. aethnensis* than in *S. chrysanthemifolius* and a previous study recorded clinal allelic variation at *Ray2* across the hybrid zone on Mount Etna (Chapman and Abbott 2010). In the present study we found that the 2-LOD confidence interval of ray area, fruit length and pappus length QTLs overlapped the *Ray2a* locus (Fig. 1). Thus, identification of *Ray2a* as an outlier locus adds to the evidence that its function in influencing capitulum size and display in *Senecio* is under divergent selection in the hybrid zone. Field studies investigating the fitness consequences of different capitulum display and fruit size at different elevations on Mount Etna are required to better understand the selective importance of this trait difference between the two species.

The EC402 gene on linkage group AC8A co-located with the 2-LOD confidence intervals of QTLs affecting pedicel length, ray area, and pappus length (Fig. 1). This gene is known to encode a ribosomal protein with possible influence on the expression of many genes and in *A. thaliana* affects responses to cold temperature, an environmental factor that varies greatly across the altitudinal gradient on Mount Etna.

The EC733 gene on linkage group AC3 (Fig. 1) encodes an ATP synthase subunit involved in hydrogen ion transport and enables a response to salt stress in *A. thaliana*. Salt concentration is unlikely to vary in a consistent manner across the range of *S. aethnensis* and *S. chrysanthemifolius,* but other ionic compounds probably vary in soils on Mount Etna according to their proximity to the active volcanic peak. No QTLs were associated with this gene, although salt stress was not examined. The only EST locus identified as significantly convergent according to the Bayesian selection analysis was ES4 in linkage group AC10A (Fig. 1). This gene is expressed in pollen with unknown function. Its status as a convergent outlier locus suggests it has an important functional role in reproduction in both species. This marker overlaps with the 2-LOD intervals of QTLs affecting several traits including capitulum number, leaf dissection, ray area and node length.

References

Alcazar R, Garcia AV, Kronholm I, de Meaux J, Koorneef M, Parker JE, Reymond M. 2010. Natural variation at Strubbelig Receptor Kinase 3 drives immune triggered incompatibilities between *Arabidopsis thaliana* accessions. *Nature Genetics* 42: 1135–1139.

Anderson EC, Slatkin M. 2003. Orr's quantitative trait loci sign test under conditions of trait ascertainment. *Genetics* 165: 445-446.

Bikard D, Patel D, Le Metté C, Giorgi V, Camilleri C, Bennett MJ, Loudet O. 2009. Divergent evolution of duplicate genes leads to genetic incompatibilities within *A. thaliana*. *Science* 323: 623–626.

Bomblies K, Weigel D. 2007. Hybrid necrosis: autoimmunity as a potential gene-flow barrier in plant species. *Nature Reviews Genetics* 8: 382–393.

Bouck A, Vision T. 2007. The molecular ecologist's guide to expressed sequence tags. *Molecular Ecology* 16: 907-924.

Brennan AC, Hiscock SJ, Abbott RJ. 2014. Interspecific crossing and genetic mapping reveal intrinsic genomic incompatibility between two *Senecio* species that form a hybrid zone on Mount Etna, Sicily. *Heredity* 113: 195-204.

Brennan AC, Bridle JR, Wang A-L, Hiscock SJ, Abbott RJ. 2009. Adaptation and selection in the Senecio (Asteraceae) hybrid zone on Mount Etna, Sicily. *New Phytologist* 183: 702-717.

Chakravarti A, Lasher LK, Reefer JE. 1991. A maximum likelihood method for estimating genome length using genetic linkage data. *Genetics* 128: 175–182.

Chapman MA, Abbott RJ. 2010. Introgression of fitness genes across a ploidy barrier. *New Phytologist* 186: 63-71.

Churchill GA, Doerge RW. 1994. Empirical threshold values for quantitative trait mapping. *Genetics* 138: 963-971.

Fishman L, Kelly AJ, Morgan E, Willis JH. 2001. A genetic map in the *Mimulus guttatus* species complex reveals transmission ratio distortion due to heterospecific interactions. *Genetics* 159: 1701-1716.

Foll M, Gaggiotti O. 2008. A genome-scan method to identify selected loci appropriate for both dominant and codominant markers: a bayesian perspective. *Genetics* 180: 977-998.

Galindo J, Grahame JW, Butlin RK. 2010. An EST-based genome scan using 454 sequencing in the marine snail *Littorina saxatilis*. *Journal of Evolutionary Biology* 23: 2004–2016.

Hegarty MJ, Barker GL, Brennan AC, Edwards KJ, Abbott RJ, Hiscock SJ. 2009. Extreme changes to gene expression associated with homoploid hybrid speciation. *Molecular Ecology* 18: 877-889.

Kim M, Cui M-L, Cubas P, Gillies A, Lee K, Chapman MA, Abbott RJ, Coen E. 2008. Regulatory genes control a key morphological and ecological trait transferred between species. *Science* 322: 1116-1119.

Lexer C, Rosenthal DM, Raymond O, Donovan LA, Rieseberg LH. 2005. Genetics of species differences in wild annual sunflowers, *Helianthus annuus* and *H. petiolaris*. *Genetics* 169: 2225–2239.

Moyle LC, Graham EB. 2006. Genome-wide associations between hybrid sterility QTL and marker transmission ratio distortion. *Molecular Biology and Evolution* 23: 973-980.

Orr HA. 1996. Dobzhansky, Bateson, and the genetics of speciation. *Genetics* 144: 1331–1335.

Orr HA. 1998. Testing natural selection vs. genetic drift in phenotypic evolution using quantitative trait locus data. *Genetics* 149: 2099-2104.

Pannell JR, Dorken ME, Eppley SM. 2005. “Haldane’s Sieve” in a metapopulation: sifting through plant reproductive polymorphisms. *Trends in Ecology and Evolution* 20: 374-379.

Rieseberg LH, Widmer A, Arntz AM, Burke JM. 2002. Directional selection is the primary cause of phenotypic diversification. *Proceedings of the National Academy of Sciences* 99: 12242–12245.

Stinchcombe JR, Hoekstra HE. 2008. Combining population genomics and quantitative genetics: finding the genes underlying ecologically important traits. *Heredity* 100: 158-170.

Van Ooijen JW. 2001. *JoinMap v3.0 Software for the calculation of genetic linkage maps.* Plant Research International, Wageningen, Netherlands. http://www.joinmap.nl

Voorrips RE. 2002. MapChart: Software for the graphical presentation of linkage maps and QTLs. *Journal of Heredity* 93: 77-78. www.biometris.nl

Zeng Z-B. 1994. Precision mapping of quantitative trait loci. *Genetics* 136: 1457-1468.
