# Supplementary material for: Genomic architecture of phenotypic divergence between two hybridizing plant species along an elevational gradient
Source: AoB Plants. 2015 Aug 18;8:plw022. doi: 10.1093/aobpla/plw022 (PMC4887755; doi:10.1093/aobpla/plw022)
Supplement: 1504_1_supp_1_nzrs7h [file 1504_1_supp_1_nzrs7h.pdf]

**Table S1.** Information on wild sampled populations of *S. aethnensis* and *S. chrysanthemifolius*.

| Species | Population name (sample size) | Latitude | Longitude | Altitude (mas) | Collector/Reference |
|---|---|---|---|---|---|
| *S. aethnensis* | PIC1 (21) | 37' 43.042 | 15' 00.060 | 2478 | ACB, Pop 7 in Brennan *et al.* 2009 |
| | PRO2 (21) | 37' 47.255 | 15' 01.201 | 2181 | ACB, Pop 14 in Brennan *et al.* 2009 |
| *S. chrysanth-emifolius* | NIC1 (14) | 37' 37.778 | 15' 01.427 | 814 | ACB and RICR, Pop 1 in Brennan *et al.* 2009 |
| | ADR2 (14) | 37' 40.201 | 14' 50.259 | 635 | ACB and RICR, Pop 8 in Brennan *et al.* 2009 |
| | RAN1 (14) | 37' 52.578 | 14' 57.390 | 658 | ACB and RICR, 2007 |

**Legend to Table S1.** Population locations and altitudes were determined using a GPS device. Altitude units are in metres above sea level. Collectors were: ACB = Adrian Brennan, RICR = Rebecca Ross. Cited article is: Brennan AC, Bridle JR, Wang A-L, Hiscock SJ, Abbott RJ (2009) Adaptation and selection in the *Senecio* (Asteraceae) hybrid zone on Mount Etna, Sicily. *New Phytologist*, 183, 702-717.

**Table S2.** Summary quantitative trait results for *S. aethnensis*, *S. chrysanthemifolius*, and a reciprocal $F_2$ *S. aethnensis* and *S. chrysanthemifolius* mapping family.

| Trait | S. aeth. mean (n, sd) | S. chrys. mean (n, sd) | F2AC mean (n, sd) | Species ratio (A/C) | F2AC mean type | QTL analysis |
|---|---|---|---|---|---|---|
| 1: Time from first true leaf to flowering (days) | 94.58 (31, 14.07) | 91.38 (32, 8.06) | 88.38 (99, 9.14) | 1.04 (ns) | chrys-like | yes |
| 2: Primary stem height (cm) | 34.22 (31, 8.01) | 43.08 (32, 6.95) | 34.92 (99, 6.29) | 0.79 | aeth-like | no, c |
| 3: Primary stem leaf number (count) | 34.87 (31, 4.84) | 37.59 (32, 6.12) | 33.88 (99, 5.43) | 0.93 (ns) | aeth-like | no, c |
| 4: Primary stem branch number (count) | 8 (32, 4.68) | 18.91 (32, 8.17) | 18.7 (100, 8.23) | 0.42 | chrys-like | no, c |
| 5: Primary stem midleaf area (mm2) | 1285.82 (31, 449.51) | 1673.65 (32, 737.61) | 1803.93 (100, 667.86) | 0.77 | chrys-like | no, c |
| 6: Primary stem midleaf perimeter (mm) | 221.59 (32, 51.36) | 1013.46 (32, 256.01) | 549.6 (100, 198.16) | 0.22 | intermediate | no, c |
| 7: Primary stem midleaf auricle width (mm) | 6.04 (31, 1.89) | 2.55 (32, 1.03) | 3.8 (99, 1.41) | 2.36 | intermediate | yes |
| 8: Primary inflorescence capitula number (count) | 3.42 (31, 1.43) | 11.47 (32, 3.56) | 9.53 (100, 3.48) | 0.3 | intermediate | yes |

| | | | | | | |
|---|---|---|---|---|---|---|
| 9: Primary capitulum pedicel length (cm) | 2.02 (32, 0.65) | 1.34 (32, 0.54) | 1.36 (100, 0.52) | 1.51 | chrys-like | yes |
| 10: Primary capitulum length (cm) | 1.14 (32, 0.12) | 0.9 (32, 0.09) | 0.94 (99, 0.09) | 1.26 | chrys-like | no, n |
| 11: Primary capitulum disc diameter (cm) | 1.12 (32, 0.15) | 0.87 (32, 0.1) | 0.87 (100, 0.12) | 1.29 | chrys-like | yes |
| 12: Primary capitulum ray number (count) | 12.66 (32, 0.6) | 13.16 (31, 0.78) | 12.8 (97, 0.59) | 0.96 | aeth-like | no, n |
| 13: Primary capitulum ray length (mm) | 12.98 (32, 2.18) | 8.26 (32, 1.58) | 9.65 (100, 1.5) | 1.57 | intermediate | no, c |
| 14: Primary capitulum ray width (mm) | 4.04 (32, 0.56) | 2.49 (31, 0.4) | 2.9 (99, 0.4) | 1.62 | intermediate | no, c |
| 15: Mean pollen number (per 3/40 florets) | 104.85 (31, 44.19) | 56.4 (32, 28.31) | 46.92 (96, 20.35) | 1.86 | chrys-like | yes |
| 16: Mean poor pollen (proportion) | 0.4 (31, 0.09) | 0.35 (32, 0.11) | 0.39 (97, 0.14) | 1.15 | no difference | yes |
| 17: Mean florets per capitulum (count) | 112.65 (32, 16.98) | 102.82 (32, 11.52) | 99.25 (99, 11.31) | 1.1 | chrys-like | no, c |
| 18: Mean selfed fruits per capitulum (count) | 0 (30, 0) | 0 (31, 0) | 0 (97, 0.01) | 0.57 (ns) | no difference | no, n |

| | | | | | | |
|---|---|---|---|---|---|---|
| 19: Mean fruit length (mm) | 3.41 (31, 0.23) | 2.35 (32, 0.23) | 2.78 (97, 0.24) | 1.46 | intermediate | yes |
| 20: Mean pappus length (mm) | 6.38 (31, 0.65) | 5.31 (32, 0.52) | 5.49 (96, 0.54) | 1.2 | chrys-like | yes |
| 21: Primary stem nodelength; height to leaf number ratio (cm) | 0.99 (32, 0.29) | 1.16 (32, 0.18) | 1.04 (100, 0.21) | 0.86 | aeth-like | yes |
| 22: Branch number to leaf number (proportion) | 0.26 (32, 0.15) | 0.54 (32, 0.25) | 0.59 (100, 0.25) | 0.49 | chrys-like | yes |
| 23: Primary capitulum size (mm3) | 4.64 (32, 1.57) | 2.19 (32, 0.67) | 2.32 (100, 0.76) | 2.12 | chrys-like | no, c |
| 24: Primary capitulum ray display area (mm2) | 166.68 (32, 44.92) | 64.81 (32, 21.14) | 88.87 (99, 23.34) | 2.57 | intermediate | yes |
| 25: Primary stem midleaf dissection; perimeter to area ratio (per mm) | 0.17 (31, 0.03) | 0.66 (32, 0.16) | 0.32 (99, 0.1) | 0.26 | intermediate | yes |

**Legend to Table S2.** Columns labelled S. aeth., S. chrys. and F2AC show the means, sample sizes (n), and standard deviations (sd) of quantitative traits for *S. aethnensis*, *S. chrysanthemifolius*, and mapping family samples, respectively. Species ratio indicates the ratio of the *S. aethnensis* to *S. chrysanthemifolius* trait means. All species trait comparisons were significantly different at a single test 95% confidence level (parametric and non-parametric tests) except for species ratios indicated by (ns). F2AC type indicates significant deviations of mapping family trait values towards either: *S. aethnensis* (aeth-like), *S. chrysanthemifolius* (chrys-like), intermediate, or not significantly different according to Tukey’s honest significant tests at a 95% adjusted confidence level. QTL analysis indicates if trait was included in QTL analysis or not. "No, c" indicates that the reason for exclusion was trait correlations or forming part of another compound trait and "no, n" indicates that the reason for exclusion was a highly skewed trait distribution.

**Table S3.** Paired trait correlations in: (a) $F_2$AC progeny; (b) *Senecio aethnensis*; (c) *S. chrysanthemifolius*; and (d) all three samples.

| (a) | 1 | 2 | 3 | 4 | 5 | 6 | 7 | 8 | 9 | 10 | 11 | 12 | 13 | 14 | 15 | 16 | 17 | 18 | 19 | 20 | 21 | 22 | 23 | 24 | 25 |
|---|---|---|---|---|---|---|---|---|---|---|---|---|---|---|---|---|---|---|---|---|---|---|---|---|---|
| 1 | - | 0.15 | 0 | 0 | 0.41 | 0 | 0.1 | 0 | 0.03 | 0.9 | 0.64 | 0.9 | 0.73 | 0.21 | 0.73 | 0.06 | 0.45 | 0.45 | 0.29 | 0.82 | 0 | 0 | 0.89 | 0.5 | 0.03 |
| 2 | 0.15 | - | 0 | 0.39 | 0.01 | 0 | 0.92 | 0 | 0.33 | 0.79 | 0.66 | 0.03 | 0.45 | 0.84 | 0.42 | 0.98 | 0 | 0.81 | 0.11 | 0.01 | 0 | 0.73 | 0.7 | 0.73 | 0.21 |
| 3 | 0.6 | 0.3 | - | 0.27 | 0.94 | 0.14 | 0.99 | 0.56 | 0.79 | 0.25 | 0.14 | 0.6 | 0.38 | 0.15 | 0.66 | 0.7 | 0.35 | 0.6 | 0.08 | 0.06 | 0 | 0.02 | 0.32 | 0.24 | 0.03 |
| 4 | -0.43 | 0.09 | 0.11 | - | 0.03 | 0.02 | 0.05 | 0.04 | 0.18 | 0.13 | 0.14 | 0.9 | 0.33 | 0.81 | 0.2 | 0.11 | 0.17 | 0.22 | 0.06 | 0.31 | 0.94 | 0 | 0.09 | 0.58 | 0.83 |
| 5 | -0.08 | 0.27 | -0.01 | 0.22 | - | 0 | 0.06 | 0.08 | 0.26 | 0.72 | 0.59 | 0.71 | 0.44 | 0.03 | 0.76 | 0.45 | 0.19 | 0.31 | 0.14 | 0.01 | 0.03 | 0.01 | 0.77 | 0.09 | 0 |
| 6 | -0.29 | 0.35 | -0.15 | 0.22 | 0.64 | - | 0.62 | 0 | 0.02 | 0.15 | 0.38 | 0.41 | 0.22 | 0.19 | 0.54 | 0.44 | 0.29 | 0.69 | 0.48 | 0.8 | 0 | 0 | 0.36 | 0.28 | 0 |
| 7 | 0.16 | -0.01 | 0 | -0.19 | 0.19 | -0.05 | - | 0.19 | 0.67 | 0.08 | 0.16 | 0.09 | 0.37 | 0.11 | 0.35 | 0.97 | 0.94 | 0.1 | 0.31 | 0.51 | 0.93 | 0.1 | 0.15 | 0.2 | 0 |
| 8 | -0.32 | 0.43 | 0.06 | 0.2 | 0.18 | 0.45 | -0.13 | - | 0.99 | 0.08 | 0.46 | 0.31 | 0.01 | 0.01 | 0.05 | 0.57 | 0 | 0.31 | 0.84 | 0.87 | 0 | 0.04 | 0.3 | 0.01 | 0 |
| 9 | -0.21 | -0.1 | -0.03 | 0.14 | -0.11 | -0.24 | -0.04 | 0 | - | 0.05 | 0.01 | 0.09 | 0.01 | 0.05 | 0.54 | 0.06 | 0.67 | 0.72 | 0.03 | 0.23 | 0.51 | 0.15 | 0.02 | 0.02 | 0.04 |
| 10 | -0.01 | 0.03 | 0.12 | 0.15 | -0.04 | -0.14 | 0.18 | -0.18 | 0.2 | - | 0 | 0.17 | 0 | 0 | 0.52 | 0.73 | 0.17 | 0.25 | 0.02 | 0.01 | 0.95 | 0.23 | 0 | 0 | 0.13 |
| 11 | 0.05 | 0.04 | 0.15 | 0.15 | 0.05 | -0.09 | 0.14 | -0.07 | 0.25 | 0.58 | - | 0.01 | 0 | 0 | 0.79 | 0.6 | 0.03 | 0.65 | 0.78 | 0.01 | 0.64 | 0.38 | 0 | 0 | 0.03 |
| 12 | -0.01 | -0.22 | 0.05 | -0.01 | 0.04 | -0.09 | 0.18 | -0.11 | 0.17 | 0.14 | 0.26 | - | 0.01 | 0.02 | 0.34 | 0.88 | 0.4 | 0.49 | 0.76 | 0.71 | 0.08 | 0.73 | 0.01 | 0.01 | 0.02 |
| 13 | 0.03 | -0.08 | 0.09 | 0.1 | 0.08 | -0.12 | 0.09 | -0.26 | 0.27 | 0.65 | 0.61 | 0.25 | - | 0 | 0.62 | 0.69 | 0.1 | 0.04 | 0.38 | 0 | 0.46 | 0.28 | 0 | 0 | 0 |
| 14 | 0.13 | -0.02 | 0.15 | -0.02 | 0.22 | -0.13 | 0.16 | -0.25 | 0.2 | 0.44 | 0.63 | 0.23 | 0.68 | - | 0.06 | 0.8 | 0.4 | 0.58 | 0.02 | 0 | 0.27 | 0.41 | 0 | 0 | 0 |
| 15 | 0.04 | -0.08 | 0.05 | -0.13 | -0.03 | -0.06 | 0.1 | -0.2 | 0.06 | 0.07 | -0.03 | 0.1 | 0.05 | 0.2 | - | 0 | 0.23 | 0.03 | 0.69 | 0.22 | 0.23 | 0.18 | 0.97 | 0.26 | 0.22 |
| 16 | 0.2 | 0 | 0.04 | -0.16 | 0.08 | -0.08 | 0 | -0.06 | -0.19 | 0.04 | -0.05 | -0.02 | -0.04 | -0.03 | -0.35 | - | 0.97 | 0.54 | 0.4 | 0.12 | 0.59 | 0.09 | 0.58 | 0.62 | 0.08 |
| 17 | 0.08 | 0.34 | 0.1 | 0.14 | 0.13 | 0.11 | -0.01 | 0.29 | 0.04 | -0.14 | 0.22 | 0.09 | -0.17 | -0.09 | -0.12 | 0 | - | 0.27 | 0.25 | 0.73 | 0.07 | 0.4 | 0.11 | 0.1 | 0.8 |
| 18 | 0.08 | -0.03 | -0.05 | -0.12 | -0.1 | -0.04 | -0.17 | -0.1 | 0.04 | 0.12 | 0.05 | -0.07 | 0.21 | 0.06 | 0.22 | 0.06 | -0.11 | - | 0.88 | 0.27 | 0.93 | 0.32 | 0.64 | 0.22 | 0.73 |

| | | | | | | | | | | | | | | | | | | | | | | | | | |
|---|---|---|---|---|---|---|---|---|---|---|---|---|---|---|---|---|---|---|---|---|---|---|---|---|---|
| 19 | -0.11 | 0.16 | 0.18 | 0.19 | 0.15 | -0.07 | 0.1 | -0.02 | 0.22 | 0.25 | 0.03 | 0.03 | 0.09 | 0.24 | 0.04 | 0.09 | -0.12 | 0.02 | - | 0 | 0.86 | 0.29 | 0.43 | 0.08 | 0 |
| 20 | 0.02 | 0.26 | 0.2 | 0.11 | 0.27 | -0.03 | 0.07 | 0.02 | 0.12 | 0.28 | 0.26 | -0.04 | 0.32 | 0.47 | 0.13 | 0.16 | -0.04 | 0.11 | 0.55 | - | 0.37 | 0.79 | 0.01 | 0 | 0 |
| 21 | -0.34 | 0.66 | -0.46 | 0.01 | 0.22 | 0.44 | -0.01 | 0.4 | -0.07 | -0.01 | -0.05 | -0.18 | -0.07 | -0.11 | -0.12 | -0.06 | 0.18 | 0.01 | 0.02 | 0.09 | - | 0.09 | 0.97 | 0.47 | 0 |
| 22 | -0.63 | -0.03 | -0.23 | 0.91 | 0.26 | 0.3 | -0.17 | 0.2 | 0.15 | 0.12 | 0.09 | 0.04 | 0.11 | -0.08 | -0.14 | -0.18 | 0.09 | -0.1 | 0.11 | 0.03 | 0.17 | - | 0.19 | 0.73 | 0.56 |
| 23 | -0.01 | 0.04 | 0.1 | 0.17 | 0.03 | -0.09 | 0.15 | -0.1 | 0.23 | 0.75 | 0.96 | 0.27 | 0.65 | 0.62 | 0 | -0.06 | 0.16 | 0.05 | 0.08 | 0.27 | 0 | 0.13 | - | 0 | 0.05 |
| 24 | 0.07 | -0.04 | 0.12 | 0.06 | 0.17 | -0.11 | 0.13 | -0.27 | 0.23 | 0.59 | 0.67 | 0.26 | 0.92 | 0.9 | 0.12 | -0.05 | -0.17 | 0.13 | 0.18 | 0.39 | -0.07 | 0.03 | 0.68 | - | 0 |
| 25 | -0.22 | 0.13 | -0.22 | 0.02 | -0.45 | 0.34 | -0.33 | 0.3 | -0.21 | -0.15 | -0.22 | -0.24 | -0.29 | -0.46 | -0.13 | -0.18 | 0.03 | 0.04 | -0.33 | -0.38 | 0.29 | 0.06 | -0.2 | -0.39 | - |

| (b) | 1 | 2 | 3 | 4 | 5 | 6 | 7 | 8 | 9 | 10 | 11 | 12 | 13 | 14 | 15 | 16 | 17 | 18 | 19 | 20 | 21 | 22 | 23 | 24 | 25 |
|---|---|---|---|---|---|---|---|---|---|---|---|---|---|---|---|---|---|---|---|---|---|---|---|---|---|
| 1 | - | 0.1 | 0 | 0 | 0 | 0 | 0.07 | 0.03 | 0.61 | 0.32 | 0.7 | 0.92 | 0.02 | 0.02 | 0.52 | 0.45 | 0.04 | 0.77 | 0.85 | 0.11 | 0 | 0 | 0.51 | 0.01 | 0.01 |
| 2 | -0.3 | - | 0.83 | 0.52 | 0.03 | 0 | 0.02 | 0.25 | 0.82 | 0.78 | 0.12 | 0.05 | 0.72 | 0.87 | 0.56 | 0.48 | 0.01 | 0.12 | 0.2 | 0.75 | 0 | 0.49 | 0.19 | 0.79 | 0.19 |
| 3 | 0.61 | -0.04 | - | 0.27 | 0.4 | 0.57 | 0.67 | 0.55 | 0.03 | 0.67 | 0.24 | 0.42 | 0.34 | 0.02 | 0.75 | 0.61 | 0.82 | 0.64 | 0.27 | 0.19 | 0 | 0.02 | 0.32 | 0.13 | 0.42 |
| 4 | -0.53 | 0.12 | -0.21 | - | 0.06 | 0.09 | 0.1 | 0 | 0.31 | 0.01 | 0.75 | 0.63 | 0.02 | 0.42 | 0.02 | 0.27 | 0.04 | 0.23 | 0.24 | 0.98 | 0.13 | 0 | 0.42 | 0.07 | 0.48 |
| 5 | -0.54 | 0.39 | -0.16 | 0.34 | - | 0 | 0 | 0.02 | 0.76 | 0.18 | 0.33 | 0.27 | 0.29 | 0.48 | 0.85 | 0.94 | 0 | 1 | 0.92 | 0.78 | 0.01 | 0.05 | 0.6 | 0.25 | 0 |
| 6 | -0.5 | 0.49 | -0.11 | 0.31 | 0.93 | - | 0 | 0 | 0.38 | 0.21 | 0.24 | 0.32 | 0.44 | 0.99 | 0.62 | 0.96 | 0 | 0.87 | 0.77 | 0.52 | 0 | 0.07 | 0.55 | 0.52 | 0 |
| 7 | -0.34 | 0.41 | -0.08 | 0.3 | 0.58 | 0.55 | - | 0.26 | 0.48 | 0.63 | 0.37 | 0.62 | 0.97 | 0.7 | 0.82 | 0.63 | 0.05 | 0.48 | 0.29 | 0.4 | 0.02 | 0.1 | 0.51 | 0.94 | 0.01 |
| 8 | -0.4 | 0.22 | -0.11 | 0.54 | 0.43 | 0.51 | 0.21 | - | 0.62 | 0.13 | 0.43 | 0.36 | 0.05 | 0.5 | 0.56 | 0.55 | 0 | 0.11 | 0.13 | 0.42 | 0.14 | 0 | 0.91 | 0.12 | 0.22 |
| 9 | -0.1 | 0.04 | -0.4 | 0.18 | 0.06 | 0.16 | 0.13 | 0.09 | - | 0.46 | 0.21 | 0.81 | 0.07 | 0.7 | 0.82 | 1 | 0.97 | 0.61 | 0.64 | 0.14 | 0.04 | 0.14 | 0.2 | 0.16 | 0.94 |
| 10 | 0.19 | 0.05 | -0.08 | -0.43 | -0.25 | -0.23 | -0.09 | -0.28 | 0.13 | - | 0 | 0.5 | 0 | 0.1 | 0 | 0.1 | 0.35 | 0.52 | 0.96 | 0.12 | 0.59 | 0.03 | 0 | 0 | 0.73 |
| 11 | 0.07 | 0.29 | 0.22 | -0.06 | 0.18 | 0.21 | 0.17 | 0.15 | 0.23 | 0.56 | - | 0.02 | 0 | 0.02 | 0.01 | 0.98 | 0.01 | 0.69 | 0.43 | 0.19 | 0.41 | 0.61 | 0 | 0 | 0.05 |

| | | | | | | | | | | | | | | | | | | | | | | | | | |
|---|---|---|---|---|---|---|---|---|---|---|---|---|---|---|---|---|---|---|---|---|---|---|---|---|---|
| 12 | 0.02 | 0.36 | 0.15 | -0.09 | 0.21 | 0.18 | 0.09 | 0.17 | 0.04 | 0.12 | 0.42 | - | 0.2 | 0.46 | 0.09 | 0.88 | 0.02 | 0.56 | 0.66 | 0.44 | 0.36 | 0.48 | 0.03 | 0.25 | 0.15 |
| 13 | 0.43 | 0.07 | 0.18 | -0.4 | -0.2 | -0.14 | 0.01 | -0.35 | 0.32 | 0.62 | 0.63 | 0.23 | - | 0 | 0.04 | 0.44 | 0.35 | 0.87 | 0.95 | 0.08 | 0.85 | 0.03 | 0 | 0 | 0.89 |
| 14 | 0.41 | -0.03 | 0.4 | -0.15 | -0.13 | 0 | 0.07 | -0.12 | 0.07 | 0.3 | 0.41 | 0.13 | 0.58 | - | 0.3 | 0.5 | 0.82 | 0.28 | 0.54 | 0.38 | 0.34 | 0.27 | 0.01 | 0 | 0.65 |
| 15 | 0.12 | 0.11 | 0.06 | -0.4 | -0.04 | -0.09 | -0.04 | -0.11 | 0.04 | 0.54 | 0.44 | 0.31 | 0.37 | 0.19 | - | 0.22 | 0.96 | 0.35 | 0.34 | 0.23 | 0.79 | 0.04 | 0 | 0.05 | 0.5 |
| 16 | 0.14 | 0.13 | -0.1 | -0.2 | 0.01 | -0.01 | 0.09 | -0.11 | 0 | 0.3 | 0 | -0.03 | 0.15 | 0.13 | 0.22 | - | 0.47 | 0.24 | 0.33 | 0.24 | 0.16 | 0.4 | 0.74 | 0.33 | 0.87 |
| 17 | -0.38 | 0.46 | 0.04 | 0.36 | 0.55 | 0.58 | 0.36 | 0.54 | 0.01 | -0.17 | 0.46 | 0.42 | -0.17 | -0.04 | 0.01 | -0.14 | - | 0.33 | 0.33 | 0.59 | 0.04 | 0.07 | 0.06 | 0.47 | 0.01 |
| 18 | 0.06 | -0.29 | -0.09 | -0.23 | 0 | -0.03 | -0.14 | -0.3 | -0.1 | 0.12 | -0.08 | 0.11 | -0.03 | -0.2 | -0.18 | -0.23 | -0.18 | - | 0.91 | 0.19 | 0.23 | 0.28 | 0.82 | 0.61 | 1 |
| 19 | -0.04 | 0.24 | -0.21 | 0.22 | 0.02 | -0.06 | 0.2 | -0.29 | 0.09 | -0.01 | -0.15 | 0.08 | 0.01 | 0.11 | 0.18 | 0.18 | -0.18 | 0.02 | - | 0.69 | 0.1 | 0.17 | 0.65 | 0.61 | 0.79 |
| 20 | 0.3 | 0.06 | 0.24 | 0 | -0.05 | -0.12 | -0.16 | -0.15 | -0.27 | 0.28 | 0.24 | -0.14 | 0.32 | 0.16 | 0.22 | 0.22 | -0.1 | -0.25 | 0.08 | - | 0.77 | 0.83 | 0.11 | 0.06 | 0.55 |
| 21 | -0.56 | 0.8 | -0.59 | 0.27 | 0.47 | 0.52 | 0.43 | 0.27 | 0.36 | 0.1 | 0.15 | 0.17 | 0.03 | -0.18 | 0.05 | 0.26 | 0.37 | -0.23 | 0.3 | -0.05 | - | 0.03 | 0.47 | 0.87 | 0.09 |
| 22 | -0.62 | 0.13 | -0.41 | 0.97 | 0.35 | 0.33 | 0.3 | 0.54 | 0.27 | -0.39 | -0.09 | -0.13 | -0.39 | -0.2 | -0.37 | -0.16 | 0.33 | -0.2 | 0.26 | -0.04 | 0.39 | - | 0.36 | 0.07 | 0.45 |
| 23 | 0.12 | 0.24 | 0.18 | -0.15 | 0.1 | 0.11 | 0.12 | 0.02 | 0.23 | 0.71 | 0.97 | 0.39 | 0.7 | 0.43 | 0.52 | 0.06 | 0.34 | -0.04 | -0.08 | 0.3 | 0.13 | -0.17 | - | 0 | 0.07 |
| 24 | 0.49 | 0.05 | 0.27 | -0.32 | -0.21 | -0.12 | 0.01 | -0.29 | 0.26 | 0.58 | 0.61 | 0.21 | 0.92 | 0.81 | 0.35 | 0.18 | -0.13 | -0.1 | 0.09 | 0.34 | -0.03 | -0.33 | 0.67 | - | 0.79 |
| 25 | 0.44 | -0.24 | 0.15 | -0.13 | -0.86 | -0.66 | -0.48 | -0.23 | -0.01 | -0.06 | -0.36 | -0.27 | -0.03 | 0.08 | -0.13 | 0.03 | -0.45 | 0 | 0.05 | -0.11 | -0.31 | -0.14 | -0.33 | 0.05 | - |

| (c) | 1 | 2 | 3 | 4 | 5 | 6 | 7 | 8 | 9 | 10 | 11 | 12 | 13 | 14 | 15 | 16 | 17 | 18 | 19 | 20 | 21 | 22 | 23 | 24 | 25 |
|---|---|---|---|---|---|---|---|---|---|---|---|---|---|---|---|---|---|---|---|---|---|---|---|---|---|
| 1 | - | 0.21 | 0 | 0.03 | 0.06 | 0.08 | 0.02 | 0.04 | 0.28 | 0.46 | 0.44 | 0.45 | 0.04 | 0.49 | 0.06 | 0.1 | 0.27 | 0.9 | 0.02 | 0.74 | 0.05 | 0 | 0.52 | 0.14 | 0.12 |
| 2 | 0.23 | - | 0.01 | 0.73 | 0.13 | 0.09 | 0.09 | 0.5 | 0.66 | 0.57 | 0.55 | 0.94 | 0.85 | 0.34 | 0.37 | 0.13 | 0.03 | 0.55 | 0.88 | 0.01 | 0 | 0.22 | 0.53 | 0.49 | 0.38 |
| 3 | 0.56 | 0.48 | - | 0.95 | 0.98 | 0.86 | 0.06 | 0.8 | 0.34 | 0.93 | 0.79 | 0.54 | 0.48 | 0.56 | 0.21 | 0.47 | 0.05 | 0.99 | 0.28 | 0.66 | 0.01 | 0.12 | 0.82 | 0.94 | 0.74 |
| 4 | -0.38 | -0.06 | 0.01 | - | 0.15 | 0.19 | 0.87 | 0.12 | 0.69 | 0.8 | 0.19 | 0.12 | 0.2 | 0.88 | 0.06 | 0.71 | 0.8 | 0.82 | 0.01 | 0.1 | 0.65 | 0 | 0.27 | 0.32 | 0.27 |

| | | | | | | | | | | | | | | | | | | | | | | | | | |
|---|---|---|---|---|---|---|---|---|---|---|---|---|---|---|---|---|---|---|---|---|---|---|---|---|---|
| 5 | -0.33 | 0.27 | 0 | 0.26 | - | 0 | 0.49 | 0.2 | 0.45 | 0.14 | 0.05 | 0.07 | 0.01 | 0.21 | 0.38 | 0.27 | 0.77 | 0.6 | 0 | 0.28 | 0.07 | 0.06 | 0.05 | 0.01 | 0 |
| 6 | -0.32 | 0.31 | 0.03 | 0.24 | 0.87 | - | 0.52 | 0.03 | 0.24 | 0.56 | 0.12 | 0.23 | 0.1 | 0.34 | 0.06 | 0.9 | 0.26 | 0.54 | 0 | 0.71 | 0.04 | 0.17 | 0.17 | 0.04 | 0 |
| 7 | 0.4 | 0.3 | 0.34 | -0.03 | 0.13 | 0.12 | - | 0.2 | 0.09 | 0.43 | 0.63 | 0.69 | 0.29 | 0.66 | 0.05 | 0.56 | 0.16 | 0.32 | 0.62 | 0.45 | 0.62 | 0.38 | 0.6 | 0.66 | 0.7 |
| 8 | -0.37 | 0.12 | -0.05 | 0.28 | 0.23 | 0.39 | -0.23 | - | 0.8 | 0.93 | 0.74 | 0.51 | 0.44 | 0.32 | 0.15 | 0.99 | 0.64 | 0.15 | 0.04 | 0.11 | 0.42 | 0.25 | 0.95 | 0.99 | 0.88 |
| 9 | 0.2 | 0.08 | 0.17 | 0.07 | 0.14 | 0.21 | 0.3 | 0.05 | - | 0.07 | 0.18 | 0.07 | 0.15 | 0.02 | 0.98 | 0.21 | 0.72 | 0.64 | 0.31 | 0.32 | 0.53 | 0.88 | 0.12 | 0.09 | 0.96 |
| 10 | -0.14 | 0.1 | 0.02 | -0.05 | 0.27 | 0.11 | -0.14 | -0.02 | 0.32 | - | 0 | 0.51 | 0 | 0.92 | 0.4 | 0.49 | 0.63 | 0.73 | 0.14 | 0.13 | 0.49 | 0.89 | 0 | 0.04 | 0.03 |
| 11 | -0.14 | 0.11 | 0.05 | -0.24 | 0.35 | 0.28 | -0.09 | 0.06 | 0.24 | 0.76 | - | 0.35 | 0 | 0.25 | 0.38 | 0.71 | 0.81 | 0.64 | 0.12 | 0.18 | 0.49 | 0.35 | 0 | 0 | 0.09 |
| 12 | 0.14 | 0.01 | 0.11 | -0.28 | -0.33 | -0.22 | -0.08 | 0.12 | -0.33 | 0.12 | 0.17 | - | 0.41 | 0.69 | 0.68 | 0.51 | 0.56 | 0.69 | 0.91 | 0.22 | 0.95 | 0.03 | 0.34 | 0.61 | 0.09 |
| 13 | -0.37 | 0.03 | -0.13 | 0.23 | 0.46 | 0.3 | -0.19 | 0.14 | -0.26 | 0.52 | 0.51 | 0.15 | - | 0.01 | 0.41 | 0.73 | 0.68 | 0.82 | 0 | 0.33 | 0.38 | 0.06 | 0 | 0 | 0 |
| 14 | -0.13 | 0.18 | 0.11 | 0.03 | 0.23 | 0.18 | 0.08 | -0.18 | -0.43 | -0.02 | 0.21 | 0.08 | 0.45 | - | 0.82 | 0.23 | 0.51 | 0.33 | 0.49 | 0.5 | 0.69 | 0.86 | 0.43 | 0 | 0.26 |
| 15 | 0.33 | 0.16 | 0.23 | -0.34 | -0.16 | -0.33 | 0.35 | -0.26 | 0.01 | 0.15 | 0.16 | 0.08 | -0.15 | 0.04 | - | 0.05 | 0.03 | 0.51 | 0.13 | 0.09 | 0.37 | 0.03 | 0.35 | 0.58 | 0.87 |
| 16 | 0.29 | 0.27 | 0.13 | -0.07 | 0.2 | 0.02 | 0.11 | 0 | 0.23 | 0.13 | 0.07 | -0.12 | -0.06 | -0.22 | 0.36 | - | 0.01 | 0.84 | 0.91 | 0.08 | 0.97 | 0.67 | 0.49 | 0.68 | 0.12 |
| 17 | -0.2 | -0.38 | -0.36 | -0.05 | 0.05 | 0.2 | -0.26 | -0.09 | -0.07 | -0.09 | 0.05 | -0.11 | 0.08 | 0.12 | -0.39 | -0.46 | - | 0.17 | 0.77 | 0.16 | 0.7 | 0.61 | 0.97 | 0.47 | 0.87 |
| 18 | 0.02 | 0.11 | 0 | 0.04 | 0.1 | 0.11 | -0.19 | 0.27 | -0.09 | 0.06 | -0.09 | -0.07 | -0.04 | -0.18 | 0.12 | 0.04 | -0.25 | - | 0.98 | 0.59 | 0.47 | 0.77 | 0.85 | 0.58 | 0.89 |
| 19 | -0.41 | -0.03 | -0.2 | 0.48 | 0.55 | 0.54 | -0.09 | 0.36 | 0.19 | 0.27 | 0.28 | -0.02 | 0.53 | 0.13 | -0.27 | -0.02 | 0.05 | 0 | - | 0.36 | 0.25 | 0 | 0.1 | 0.01 | 0.04 |
| 20 | 0.06 | 0.47 | -0.08 | -0.29 | 0.2 | 0.07 | 0.14 | -0.29 | -0.18 | 0.27 | 0.24 | 0.23 | 0.18 | 0.13 | 0.31 | 0.31 | -0.25 | -0.1 | 0.17 | - | 0 | 0.24 | 0.1 | 0.35 | 0.17 |
| 21 | -0.35 | 0.49 | -0.46 | -0.08 | 0.32 | 0.36 | -0.09 | 0.15 | -0.11 | 0.13 | 0.13 | -0.01 | 0.16 | 0.07 | -0.16 | -0.01 | 0.07 | 0.14 | 0.21 | 0.58 | - | 0.77 | 0.46 | 0.38 | 0.2 |
| 22 | -0.6 | -0.22 | -0.28 | 0.91 | 0.33 | 0.25 | -0.16 | 0.21 | 0.03 | 0.03 | -0.17 | -0.4 | 0.34 | 0.03 | -0.39 | -0.08 | 0.09 | 0.06 | 0.56 | -0.22 | 0.05 | - | 0.49 | 0.19 | 0.07 |
| 23 | -0.12 | 0.11 | 0.04 | -0.2 | 0.35 | 0.25 | -0.1 | 0.01 | 0.28 | 0.88 | 0.97 | 0.18 | 0.54 | 0.15 | 0.17 | 0.13 | 0.01 | -0.04 | 0.3 | 0.3 | 0.14 | -0.13 | - | 0 | 0.05 |
| 24 | -0.26 | 0.13 | -0.01 | 0.18 | 0.48 | 0.37 | -0.08 | 0 | -0.31 | 0.36 | 0.5 | 0.1 | 0.88 | 0.79 | -0.1 | -0.08 | 0.13 | -0.1 | 0.43 | 0.17 | 0.16 | 0.24 | 0.49 | - | 0.01 |

| | | | | | | | | | | | | | | | | | | | | | | | | | |
|---|---|---|---|---|---|---|---|---|---|---|---|---|---|---|---|---|---|---|---|---|---|---|---|---|---|
| 25 | 0.28 | -0.16 | 0.06 | -0.2 | -0.87 | -0.54 | -0.07 | -0.03 | -0.01 | -0.38 | -0.3 | 0.31 | -0.5 | -0.21 | 0.03 | -0.28 | 0.03 | -0.03 | -0.37 | -0.25 | -0.23 | -0.32 | -0.35 | -0.46 | - |

| (d) | 1 | 2 | 3 | 4 | 5 | 6 | 7 | 8 | 9 | 10 | 11 | 12 | 13 | 14 | 15 | 16 | 17 | 18 | 19 | 20 | 21 | 22 | 23 | 24 | 25 |
|---|---|---|---|---|---|---|---|---|---|---|---|---|---|---|---|---|---|---|---|---|---|---|---|---|---|
| 1 | - | 0 | 1 | 1 | 0 | 0 | 0 | 0 | 0 | 0 | 0 | 0 | 0 | 0 | 0 | 0 | 0 | 0 | 0 | 0 | 0 | 1 | 0 | 0 | 0 |
| 2 | - | - | 0 | 0 | 0 | 0 | 0 | 1 | 0 | 0 | 0 | 0 | 0 | 0 | 0 | 0 | 0 | 0 | 0 | 0 | 2 | 0 | 0 | 0 | 0 |
| 3 | - | - | - | 0 | 0 | 0 | 0 | 0 | 0 | 0 | 0 | 0 | 0 | 0 | 0 | 0 | 0 | 0 | 0 | 0 | 1 | 0 | 0 | 0 | 0 |
| 4 | - | - | - | - | 0 | 0 | 0 | 0 | 0 | 0 | 0 | 0 | 0 | 0 | 0 | 0 | 0 | 0 | 0 | 0 | 0 | 3 | 0 | 0 | 0 |
| 5 | - | - | - | - | - | 3 | 0 | 0 | 0 | 0 | 0 | 0 | 0 | 0 | 0 | 0 | 0 | 0 | 0 | 0 | 0 | 0 | 0 | 0 | 3 |
| 6 | - | - | - | - | - | - | 0 | 1 | 0 | 0 | 0 | 0 | 0 | 0 | 0 | 0 | 0 | 0 | 0 | 0 | 1 | 0 | 0 | 0 | 1 |
| 7 | - | - | - | - | - | - | - | 0 | 0 | 0 | 0 | 0 | 0 | 0 | 0 | 0 | 0 | 0 | 0 | 0 | 0 | 0 | 0 | 0 | 0 |
| 8 | - | - | - | - | - | - | - | - | 0 | 0 | 0 | 0 | 0 | 0 | 0 | 0 | 0 | 0 | 0 | 0 | 1 | 0 | 0 | 0 | 0 |
| 9 | - | - | - | - | - | - | - | - | - | 0 | 0 | 0 | 0 | 0 | 0 | 0 | 0 | 0 | 0 | 0 | 0 | 0 | 0 | 0 | 0 |
| 10 | - | - | - | - | - | - | - | - | - | - | 2 | 0 | 2 | 1 | 0 | 0 | 0 | 0 | 0 | 0 | 0 | 0 | 3 | 1 | 0 |
| 11 | - | - | - | - | - | - | - | - | - | - | - | 0 | 2 | 1 | 0 | 0 | 0 | 0 | 0 | 0 | 0 | 0 | 3 | 1 | 0 |
| 12 | - | - | - | - | - | - | - | - | - | - | - | - | 0 | 0 | 0 | 0 | 0 | 0 | 0 | 0 | 0 | 0 | 0 | 0 | 0 |
| 13 | - | - | - | - | - | - | - | - | - | - | - | - | - | 1 | 0 | 0 | 0 | 0 | 0 | 0 | 0 | 0 | 2 | 3 | 0 |
| 14 | - | - | - | - | - | - | - | - | - | - | - | - | - | - | 0 | 0 | 0 | 0 | 0 | 1 | 0 | 0 | 1 | 3 | 1 |
| 15 | - | - | - | - | - | - | - | - | - | - | - | - | - | - | - | 0 | 0 | 0 | 0 | 0 | 0 | 0 | 0 | 0 | 0 |
| 16 | - | - | - | - | - | - | - | - | - | - | - | - | - | - | - | - | 0 | 0 | 0 | 0 | 0 | 0 | 0 | 0 | 0 |
| 17 | - | - | - | - | - | - | - | - | - | - | - | - | - | - | - | - | - | 0 | 0 | 0 | 0 | 0 | 0 | 0 | 0 |

| | | | | | | | | | | | | | | | | | | | | | | | | | |
|---|---|---|---|---|---|---|---|---|---|---|---|---|---|---|---|---|---|---|---|---|---|---|---|---|---|
| 18 | - | - | - | - | - | - | - | - | - | - | - | - | - | - | - | - | - | - | 0 | 0 | 0 | 0 | 0 | 0 | 0 |
| 19 | - | - | - | - | - | - | - | - | - | - | - | - | - | - | - | - | - | - | - | 1 | 0 | 0 | 0 | 0 | 0 |
| 20 | - | - | - | - | - | - | - | - | - | - | - | - | - | - | - | - | - | - | - | - | 0 | 0 | 0 | 1 | 1 |
| 21 | - | - | - | - | - | - | - | - | - | - | - | - | - | - | - | - | - | - | - | - | - | 0 | 0 | 0 | 0 |
| 22 | - | - | - | - | - | - | - | - | - | - | - | - | - | - | - | - | - | - | - | - | - | - | 0 | 0 | 0 |
| 23 | - | - | - | - | - | - | - | - | - | - | - | - | - | - | - | - | - | - | - | - | - | - | - | 2 | 0 |
| 24 | - | - | - | - | - | - | - | - | - | - | - | - | - | - | - | - | - | - | - | - | - | - | - | - | 1 |
| 25 | - | - | - | - | - | - | - | - | - | - | - | - | - | - | - | - | - | - | - | - | - | - | - | - | - |

**Legend to Table S3.** Paired trait correlations in: (a) F2AC progeny; (b) *Senecio aethnensis*; (c) *S. chrysanthemifolius*; and (d) all three samples. Trait numbers follow Table S2. For (a); (b) and (c) values below and above the left to right diagonal are spearman rank correlation coefficients and their p-values respectively. Shaded values indicate significantly correlated traits following Bonferroni correction ($p \leq 0.05/300$). For (d) numbers represent the frequency that paired trait correlations with $p \leq 0.05/300$ were observed across the three samples. Shaded columns and rows indicate traits that were excluded from the quantitative trait locus analyses due to either highly non-normal distributions; correlations with other traits; or because they were used to calculate compound traits (node length; branch to node ratio; capitulum size; ray area and leaf dissection).

**Table S4.** Comparison of summary quantitative trait locus results for a composite interval mapping (CIM) and multiple interval mapping (MIM) analysis of a reciprocal $F_2$ *S. aethnensis* and *S. chrysanthemifolius* mapping family.

| | CIM | | | | | MIM | | | | | |
|---|---|---|---|---|---|---|---|---|---|---|---|
| Trait | QTL LG and peak | QTL region (peak LOD, threshold) | Add. effect | Dom. effect | PVE | QTL LG and peak | QTL region (peak LOD) | Add. effect (PVE) | Dom. effect (PVE) | PVE | Epi-stasis |
| 1 | AC1, 3.5 | 0.9 - 3.5 (4.22, 3.15) | 13.23 | -12.41 | 16.7 | AC1, 41.3 | 36.6 - 44.5 (6.19) | -3.41 (5.7) | -2.31 (1.3) | 7 | 2x3 (5.4) |
| 1 | AC2, 8.8 | 0 - 14.2 (5.32, 3.15) | -4.67 | 2.33 | 13.4 | AC2, 9.3 | 6.3 - 11.3 (9.22) | -5.27 (14.3) | 0.13 (0) | 14.3 | |
| 1 | AC10A, 3.4 | 0 - 10 (11.4, 3.15) | 7.56 | -2.49 | 32.5 | AC10A, 0 | 0 - 0.5 (15.35) | 9.02 (46.2) | -3.56 (2.3) | 48.5 | |
| 7 | | | | | | AC10B, 3 | 0 - 4.2 (2.21) | 0.52 (7.3) | 0.41 (2.7) | 10 | |
| 8 | | | | | | AC2, 11.3 | 0 - 14.2 (2.5) | 1.29 (8.5) | 0.96 (3.6) | 12.1 | |
| 8 | AC5A, 0 | 0 - 6.9 | -1.33 | 0.75 | 8.6 | AC5A, 0 | 0 - 6.9 | -1.34 | 0.36 | 9.4 | |

| | | | | | | | | | | |
|---|---|---|---|---|---|---|---|---|---|---|
| | | (2.88, 2.74) | | | | | (2.57) | (9) | (0.4) | |
| 8 | AC10A, 0 | 0 - 10<br>(6.37, 2.74) | 2.48 | -0.8 | 22.5 | AC10A, 0 | 0 - 10<br>(4.89) | 2.14<br>(20.7) | -0.43<br>(-0.1) | 20.6 |
| 9 | AC5A, 0 | 0 - 6.9<br>(3.35, 2.94) | 0.21 | -0.2 | 11.2 | | | | | |
| 9 | | | | | | AC8A, 11.4 | 0 - 27.5<br>(2.5) | 0.26<br>(11.7) | -0.07<br>(0.4) | 12.1 |
| 11 | | | | | | AC4, 23.6 | 12.5 - 36.3<br>(3.52) | 0.05<br>(6.4) | 0.08<br>(9.8) | 16.2 |
| 11 | | | | | | AC9, 0 | 0 - 15<br>(2.13) | -0.05<br>(6.2) | 0.03<br>(1.2) | 7.4 |
| 15 | AC10B, 3 | 0 - 4.2<br>(3.52, 2.9) | 8.21 | -13.78 | 14.5 | AC10B, 3 | 0 - 4.2<br>(2.03) | 7.79<br>(6.2) | -8.94<br>(3.8) | 10 |
| 16 | AC1, 0.9 | 0 - 1<br>(7.79, 2.86) | -0.02 | -0.22 | 27.7 | AC1, 0.9 | 0 - 5.5<br>(5.4) | 0.01<br>(-1.2) | -0.19<br>(25.9) | 24.7 |
| 16 | AC8A, 16.2 | 3 - 16.3<br>(3.92, 2.86) | 0.11 | -0.05 | 11.3 | | | | | |

| | | | | | | | | | | |
|---|---|---|---|---|---|---|---|---|---|---|
| 19 | AC1, 20 | 1.4 - 37.6 (3.05, 2.84) | -0.19 | 0.32 | 13.6 | AC1, 18 | 2.4 - 39.6 (2.89) | -0.21 (-5.8) | 0.36 (26.1) | 20.3 |
| 20 | AC1, 29.9 | 18 - 37.6 (4.89, 2.87) | -0.02 | 0.52 | 22.2 | AC1, 23 | 8.5 - 36.6 (4.6) | 0.15 (5.9) | 0.32 (12) | 17.9 |
| 20 | | | | | | AC5B, 0 | 0 - 9.5 (2.09) | 0.2 (5.6) | 0.01 (0) | 5.6 |
| 20 | AC8A, 26 | 3 - 27.5 (3.57, 2.87) | 0.3 | 0.19 | 15 | AC8A, 13.6 | 0 - 27.5 (3.43) | 0.27 (10.6) | 0.12 (1.3) | 11.9 |
| 21 | AC4, 18.9 | 1.5 - 30.3 (3.68, 2.87) | 0.09 | 0.04 | 8.8 | AC4, 6 | 0 - 41.3 (2.8) | 0.09 (8.6) | 0.1 (2.9) | 11.5 |
| 21 | AC5A, 6.5 | 0 - 6.9 (6.36, 2.87) | -0.12 | 0 | 16.8 | AC5A, 0 | 0 - 6.9 (6.37) | -0.12 (18) | -0.01 (0.1) | 18.1 |
| 21 | AC10A, 4.1 | 0 - 10 (10.24, 2.87) | 0.15 | -0.08 | 24.7 | AC10A, 4.1 | 0 - 10 (11.57) | 0.16 (30.1) | -0.11 (2) | 32.1 |
| 22 | AC4, 39.3 | 14.5 - 41.3 (3.04, 2.9) | 0.13 | -0.16 | 24.2 | AC4, 26.6 | 13.5 - 41.3 (4.3) | 0.1 (9.9) | -0.17 (12.1) | 22 |
| 24 | | | | | | AC1, 15 | 6.5 - 25 | 4.69 | 3.37 | 5.2 |

| | | | | | | | | | | | |
|---|---|---|---|---|---|---|---|---|---|---|---|
| | | | | | | | (6.75) | (3.3) | (1.9) | | |
| 24 | | | | | | AC4, 30.3 | 4 - 41.3 (2.07) | 6.9 (6.2) | 0.25 (0.1) | 6.3 | |
| 24 | AC7A, 1.3 | 0 - 14.2 (3.73, 2.88) | 4.35 | 13.14 | 10.1 | AC7A, 4.6 | 0 - 14.2 (6.45) | 5.79 (2.1) | 16.83 (7.1) | 9.2 | 1x3 (5.1) |
| 24 | | | | | | AC8A, 15.1 | 2.5 - 24.5 (5.5) | 8.13 (8) | 5.67 (-0.2) | 7.8 | 1x4 (7.8) |
| 24 | AC10A, 3.4 | 0 - 8.6 (6.03, 2.88) | 12.9 | 5.57 | 18.3 | AC10A, 2.8 | 0 - 8.6 (8.01) | 15.43 (20.9) | 0.54 (0.2) | 21.1 | |
| 25 | | | | | | AC4, 3 | 0 - 41.3 (2.02) | 0.04 (6.9) | 0.04 (2.9) | 9.8 | |
| 25 | AC8A, 9 | 1.5 - 11.4 (4.86, 3.02) | 0.09 | 0.04 | 18.9 | AC8A, 6 | 0 - 10 (7.29) | 0.04 (7.7) | 0.05 (6.3) | 14 | 2x3 (10.1) |
| 25 | AC10A, 0 | 0 - 10 (5.45, 3.02) | 0.06 | 0 | 16.9 | AC10A, 0 | 0 - 10 (9.01) | 0.06 (21.1) | 0.01 (0.5) | 21.6 | |
| 25 | | | | | | AC10B, 3 | 0 - 4.2 (2.6) | 0.02 (3.9) | 0.04 (4.4) | 8.3 | |

**Legend to Table S4.** Trait numbers follow Table S2. QTLs that were identified by both analyses are listed in the same row. QTL LG and peak are the linkage group and maximum likelihood of odds score (LOD) cM position of significant QTLs identified from CIM or MIM analysis. QTL region is the cM range within a 2-LOD interval around the QTL peak with the peak LOD value and 1000 permutation 0.95 quantile LOD threshold (CIM only) indicated in parentheses. The additive (add.) and dominance (dom.) effects are in the same units as trait measures. Positive additive effects support the direction of the species difference and vice versa for negative effects; while positive dominance effects indicate that *S. aethnensis* alleles are dominant and vice versa for negative effects. The percentage variance explained (PVE) is shown in parentheses. Epistasis shows the significantly interacting loci for each trait (numbered in the order they appear in the table) with the additional PVE for the interaction shown in parentheses.

**Table S5.** Summary quantitative trait loci (QTLs) results from a multiple trait composite interval mapping (MtCIM) analysis compared to single trait QTL analyses of a reciprocal $F_2$ *S. aethnensis* and *S. chrysanthemifolius* mapping family.

| Locus LG, peak cM position | Locus 2 LOD interval (peak LOD) | Overlapping CIM QTL LOD intervals | Overlapping MIM QTL LOD intervals |
|---|---|---|---|
| AC1, 0.9 | 0 - 6.5 (14.52) | 1: Time from first true leaf to flowering,<br>16: Mean poor pollen,<br>19: Mean fruit length | 16: Mean poor pollen |
| AC1, 29.9 | 26.9 - 32.9 (14.35) | 19: Mean fruit length,<br>20: Mean pappus length | 19: Mean fruit length,<br>20: Mean pappus length |
| AC2, 5.8 | 1.5 - 11.3 (14.1) | 1: Time from first true leaf to flowering | 1: Time from first true leaf to flowering,<br>8: Primary inflorescence capitulum number |
| AC4, 3 | 1.8 - 4.2 (17.69) | 21: Primary stem node length | 21: Primary stem node length,<br>24: Primary capitulum ray display area,<br>25: Primary stem midleaf dissection |
| AC5A, 3 | 0 - 6.9 (17.24) | 8: Primary inflorescence capitulum number,<br>9: Primary capitulum pedicel length,<br>21: Primary stem node length | 8: Primary inflorescence capitulum number,<br>21: Primary stem node length |
| AC10A, 8.6 | 3.4 - 10 (28.6) | 1: Time first true leaf to flowering,<br>8: Primary inflorescence capitulum number,<br>21: Primary stem node length,<br>24: Primary capitulum ray display area,<br>25: Primary stem midleaf dissection | 8: Primary inflorescence capitulum number,<br>21: Primary stem node length,<br>24: Primary capitulum ray display area,<br>25: Primary stem midleaf dissection |

**Legend to Table S5.** Locus LG and peak cM are the linkage group and maximum likelihood odds score (LOD) cM position of a locus that affects the expression of multiple traits. Locus 2 LOD interval is the 2-LOD interval around the peak for the locus with the maximum LOD value indicated in parentheses. CIM and MIM refers to coincident QTLs identified using composite interval mapping and multiple interval mapping, respectively (see Tables 1 and S4 for more details about QTLs).

**Table S6.** (a) "Sampling without replacement" test results for paired-trait QTL coincidence (b) permutation tests of overall paired-trait QTL coincidence using different QTL and transmission ratio distortion loci (TRDL) datasets and genetic map interval sizes.

| (a) Trait | 7 | 22 | 8 | 11 | 19 | 25 | 15 | 21 | 20 | 7 | 1 | 16 | 24 | TRDL clusters | All TRDLs |
|---|---|---|---|---|---|---|---|---|---|---|---|---|---|---|---|
| 7: auricle width | - | - | - | - | - | - | - | - | - | - | - | - | - | - | - |
| 22:branch node ratio | 0.964 | - | - | - | - | - | - | - | - | - | - | - | - | - | - |
| 8: capitulum number | 0.893 | 0.893 | - | - | - | - | - | - | - | - | - | - | - | - | - |
| 11: disc diameter | 0.929 | 0.071 | 0.794 | - | - | - | - | - | - | - | - | - | - | - | - |
| 19: fruit length | 0.964 | 0.964 | 0.893 | 0.929 | - | - | - | - | - | - | - | - | - | - | - |
| 25: leaf disection | 0.143 | 0.857 | 0.337 | 0.730 | 0.857 | - | - | - | - | - | - | - | - | - | - |
| 15: pollen number | 0.036 | 0.964 | 0.893 | 0.929 | 0.964 | 0.143 | - | - | - | - | - | - | - | - | - |
| 21:node length | 0.893 | 0.893 | 0.023 | 0.794 | 0.893 | 0.044 | 0.893 | - | - | - | - | - | - | - | - |
| 20: pappus length | 0.893 | 0.893 | 0.702 | 0.794 | 0.107 | 0.337 | 0.893 | 0.702 | - | - | - | - | - | - | - |
| 7: pedicel length | 0.964 | 0.964 | 0.893 | 0.929 | 0.964 | 0.143 | 0.964 | 0.893 | 0.107 | - | - | - | - | - | - |
| 1: flowering time | 0.893 | 0.893 | 0.023 | 0.794 | 0.893 | 0.337 | 0.893 | 0.275 | 0.702 | 0.893 | - | - | - | - | - |
| 16: pollen viability | 0.964 | 0.964 | 0.893 | 0.929 | 0.964 | 0.857 | 0.964 | 0.893 | 0.893 | 0.964 | 0.893 | - | - | - | - |
| 24: ray area | 0.821 | 0.821 | 0.386 | 0.669 | 0.179 | 0.124 | 0.821 | 0.386 | 0.070 | 0.179 | 0.386 | 0.821 | - | - | - |
| TRDL clusters | 0.857 | 0.857 | 0.337 | 0.730 | 0.857 | 0.395 | 0.857 | 0.337 | 0.618 | 0.857 | 0.337 | 0.143 | 0.124 | - | - |
| All TRDLs | 0.679 | 0.679 | 0.470 | 0.452 | 0.679 | 0.301 | 0.679 | 0.209 | 0.296 | 0.679 | 0.470 | 0.321 | 0.355 | - | - |

| (b) Included traits | Mean cM interval size | Permutations | Mean obs. p value (st. dev.) | Mean null p value (st. dev.) | Probability of non-coincidence |
|---|---|---|---|---|---|
| QTLs only | mean (16.5) | 1000 | 0.694 (0.326) | 0.752 (0.024) | 0.020 |
| QTLs and all TRDLs | mean (16.5) | 1000 | 0.664 (0.317) | 0.709 (0.023) | 0.043 |
| QTLs and TRDL clusters | mean (16.5) | 1000 | 0.675 (0.323) | 0.734 (0.022) | 0.012 |
| QTLs only | 2 | 100 | 0.89 (0.269) | 0.947 (0.017) | 0.010 |
| QTLs only | 4 | 100 | 0.815 (0.327) | 0.906 (0.021) | 0.010 |
| QTLs only | 6 | 100 | 0.805 (0.306) | 0.869 (0.022) | 0.030 |
| QTLs only | 8 | 100 | 0.786 (0.317) | 0.857 (0.023) | 0.020 |
| QTLs only | 10 | 100 | 0.748 (0.321) | 0.818 (0.022) | 0.010 |
| QTLs only | 12 | 100 | 0.739 (0.329) | 0.815 (0.023) | 0.010 |
| QTLs only | 14 | 100 | 0.735 (0.321) | 0.797 (0.024) | 0.030 |
| QTLs only | 16 | 100 | 0.694 (0.326) | 0.753 (0.026) | 0.030 |

**Legend to Table S6.** (a) Column and row names indicate the paired-trait quantitiative trait loci (QTL) combinations for each table entry. Trait numbers follow Table S2. TRDL clusters are genomic regions with multiple transmission ratio distortion loci separated by < 10 cM map distance. All TRDLs are transmission ratio distortion loci whose positions were defined by the most significantly distorted locus in a

cluster of distorted loci. QTLs and their 2-LOD intervals identified from multiple interval mapping (MIM) were used and the mean 2-LOD QTL confidence range (16.5 cM) was used as the genetic map interval bin size used to build the QTL (or TRDL) occupancy tables. Presented values are p values for non-coincidence with values less than 0.05 indicating significantly coincident paired-trait QTLs.

(b) Included traits indicate if permutation tests were based on: QTLs only, QTLs and all TRDLs, or QTLs and TRDL clusters (distorted regions comprising more than one marker). Mean cM interval size indicates the genetic map interval size used to build the QTL (TRDL) occupancy tables. Permutations is the number of randomized occupancy tables tested. Mean obs. p value (st. dev.) is the observed mean (and standard deviation) p value for paired-trait non-coincidence across all paired traits examined. Mean null p value (st. dev.) is the permutation mean of the mean (and standard deviation) p value for paired-trait non-coincidence across all paired traits examined. Probability of non-coincidence is the permutation-based probability of accepting the null hypothesis that the observed mean could be explained by a random distribution of QTL or TRDL locations.

**Table S7.** Summary population genetic statistics for AFLPs and dominant scored molecular genetic markers from *S. aethnensis* and *S. chrysanthemifolius* samples.

| Locus | *N* | Band Freq. | *p* | $N_e$ | $UH_e$ | *Ar* | *pAr* | $F_{ST}$ | $Phi_{PT}$ |
|---|---|---|---|---|---|---|---|---|---|
| E1M3_104 | 36, 38 | 0.78, 0.48 | 0.53, 0.28 | 1.99, 1.67 | 0.51, 0.41 | 2, 2 | 0, 0 | 0.1 | 0.09* |
| E1M3_118 | 36, 38 | 0.44, 0.06 | 0.26, 0.03 | 1.61, 1.06 | 0.37, 0.06 | 2, 1.98 | 0.02, 0 | 0.21 | 0.21* |
| E1M3_121 | 36, 38 | 0.31, 0.16 | 0.17, 0.09 | 1.39, 1.2 | 0.29, 0.15 | 2, 2 | 0, 0 | 0.03 | 0.02 |
| E1M3_124 | 36, 38 | 0.22, 0 | 0.12, 0 | 1.27, 1 | 0.21, 0 | 2, 1 | 1, 0 | 0.13 | 0.1* |
| E1M3_126 | 36, 38 | 0.28, 0.63 | 0.15, 0.43 | 1.35, 1.68 | 0.26, 0.41 | 2, 2 | 0, 0 | 0.13 | 0.13* |
| E1M3_145 | 36, 38 | 0.08, 0.06 | 0.04, 0.03 | 1.09, 1.06 | 0.08, 0.06 | 2, 1.98 | 0.02, 0 | 0 | -0.01 |
| E1M3_146 | 36, 38 | 0, 0.26 | 0, 0.14 | 1, 1.33 | 0, 0.24 | 1, 2 | 0, 1 | 0.15 | 0.09* |
| E1M3_147 | 36, 38 | 0.89, 0.37 | 0.68, 0.21 | 1.75, 1.5 | 0.43, 0.33 | 2, 2 | 0, 0 | 0.29 | 0.29* |
| E1M3_149 | 36, 38 | 0.31, 0.73 | 0.17, 0.52 | 1.39, 1.72 | 0.29, 0.43 | 2, 2 | 0, 0 | 0.19 | 0.2* |
| E1M3_163 | 36, 38 | 0.06, 0.57 | 0.03, 0.36 | 1.06, 1.79 | 0.06, 0.45 | 1.99, 2 | 0, 0.01 | 0.32 | 0.3* |
| E1M3_175 | 36, 38 | 0.25, 0.52 | 0.13, 0.31 | 1.3, 1.74 | 0.24, 0.44 | 2, 2 | 0, 0 | 0.08 | 0.07* |
| E1M3_177 | 36, 38 | 0.39, 0.2 | 0.22, 0.11 | 1.52, 1.26 | 0.35, 0.19 | 2, 2 | 0, 0 | 0.04 | 0.03 |
| E1M3_179 | 36, 38 | 0.22, 0.75 | 0.12, 0.59 | 1.26, 1.59 | 0.21, 0.33 | 2, 2 | 0, 0 | 0.27 | 0.28* |
| E1M3_190 | 36, 38 | 0.58, 0 | 0.39, 0 | 1.68, 1 | 0.4, 0 | 2, 1 | 1, 0 | 0.41 | 0.4* |

| | | | | | | | | | |
|---|---|---|---|---|---|---|---|---|---|
| E1M3_196 | 36, 38 | 0, 0.13 | 0, 0.07 | 1, 1.15 | 0, 0.13 | 1, 2 | 0, 1 | 0.07 | 0.01 |
| E1M3_197 | 36, 38 | 1, 0.62 | 1, 0.39 | 1, 1.85 | 0, 0.48 | 1, 2 | 0, 1 | 0.23 | 0.18* |
| E1M3_206 | 36, 38 | 0.25, 0.73 | 0.13, 0.49 | 1.3, 1.92 | 0.24, 0.5 | 2, 2 | 0, 0 | 0.24 | 0.25* |
| E1M3_209 | 36, 38 | 0.03, 0.78 | 0.01, 0.62 | 1.03, 1.61 | 0.03, 0.33 | 1.99, 2 | 0, 0.01 | 0.6 | 0.54* |
| E1M3_210 | 36, 38 | 0.94, 0.08 | 0.83, 0.04 | 1.4, 1.09 | 0.23, 0.08 | 2, 2 | 0, 0 | 0.75 | 0.65* |
| E1M3_216 | 36, 38 | 0.81, 0.16 | 0.57, 0.09 | 1.9, 1.2 | 0.48, 0.16 | 2, 2 | 0, 0 | 0.42 | 0.42* |
| E1M3_219 | 36, 38 | 0.81, 1 | 0.56, 1 | 1.96, 1 | 0.5, 0 | 2, 1 | 1, 0 | 0.11 | 0.08* |
| E1M3_222 | 36, 38 | 0.56, 0.5 | 0.34, 0.29 | 1.78, 1.71 | 0.45, 0.43 | 2, 2 | 0, 0 | 0 | -0.02 |
| E1M3_254 | 36, 38 | 0.83, 0.29 | 0.62, 0.16 | 1.78, 1.37 | 0.44, 0.28 | 2, 2 | 0, 0 | 0.3 | 0.31* |
| E1M3_264 | 36, 38 | 1, 0.6 | 1, 0.38 | 1, 1.8 | 0, 0.46 | 1, 2 | 0, 1 | 0.25 | 0.2* |
| E1M3_294 | 36, 38 | 0.94, 0 | 0.76, 0 | 1.56, 1 | 0.37, 0 | 2, 1 | 1, 0 | 0.89 | 0.73* |
| E1M3_303 | 36, 38 | 0, 0 | 0, 0 | 1, 1 | 0, 0 | 1, 1 | 0, 0 | n/a | n/a |
| E1M3_317 | 36, 38 | 0, 0.29 | 0, 0.16 | 1, 1.37 | 0, 0.28 | 1, 2 | 0, 1 | 0.17 | 0.11* |
| E1M3_57 | 35, 37 | 0.09, 0.33 | 0.04, 0.18 | 1.09, 1.43 | 0.09, 0.31 | 2, 2 | 0, 0 | 0.09 | 0.05* |
| E1M3_70 | 36, 38 | 0.08, 0.87 | 0.04, 0.71 | 1.09, 1.63 | 0.08, 0.34 | 2, 2 | 0, 0 | 0.62 | 0.56* |
| E1M3_76 | 36, 38 | 0.14, 0.75 | 0.07, 0.6 | 1.16, 1.57 | 0.14, 0.32 | 2, 2 | 0, 0 | 0.39 | 0.39* |
| E1M3_97 | 36, 38 | 0.94, 0.73 | 0.83, 0.49 | 1.4, 1.92 | 0.23, 0.5 | 2, 2 | 0, 0 | 0.08 | 0.04 |

| | | | | | | | | | |
|---|---|---|---|---|---|---|---|---|---|
| E1M3_98 | 36, 38 | 0, 0 | 0, 0 | 1, 1 | 0, 0 | 1, 1 | 0, 0 | n/a | n/a |
| E1M5_125 | 37, 40 | 0.97, 0.22 | 0.88, 0.13 | 1.28, 1.3 | 0.19, 0.18 | 2, 2 | 0, 0 | 0.58 | 0.58* |
| E1M5_131 | 37, 40 | 0.35, 0.73 | 0.2, 0.59 | 1.47, 1.52 | 0.3, 0.3 | 2, 2 | 0, 0 | 0.16 | 0.2* |
| E1M5_132 | 37, 40 | 0.94, 0.73 | 0.83, 0.49 | 1.4, 1.92 | 0.23, 0.5 | 2, 2 | 0, 0 | 0.09 | 0.07* |
| E1M5_138 | 37, 40 | 0.11, 0.68 | 0.06, 0.44 | 1.12, 1.94 | 0.11, 0.5 | 2, 2 | 0, 0 | 0.34 | 0.37* |
| E1M5_140 | 37, 40 | 0.05, 0 | 0.03, 0 | 1.06, 1 | 0.05, 0 | 1.98, 1 | 0.98, 0 | n/a | n/a |
| E1M5_150 | 37, 40 | 0, 0.34 | 0, 0.2 | 1, 1.46 | 0, 0.29 | 1, 2 | 0, 1 | 0.21 | 0.19* |
| E1M5_160 | 37, 40 | 0.75, 0.64 | 0.51, 0.42 | 1.94, 1.79 | 0.5, 0.45 | 2, 2 | 0, 0 | 0.01 | 0 |
| E1M5_168 | 37, 40 | 0.57, 0.33 | 0.37, 0.18 | 1.74, 1.42 | 0.42, 0.3 | 2, 2 | 0, 0 | 0.06 | 0.07* |
| E1M5_169 | 37, 40 | 0.57, 0.69 | 0.36, 0.55 | 1.78, 1.51 | 0.44, 0.3 | 2, 2 | 0, 0 | 0.02 | 0.01 |
| E1M5_202 | 37, 40 | 0.89, 0.48 | 0.67, 0.28 | 1.79, 1.66 | 0.45, 0.4 | 2, 2 | 0, 0 | 0.2 | 0.22* |
| E1M5_204 | 37, 40 | 0.92, 0.05 | 0.72, 0.03 | 1.67, 1.05 | 0.41, 0.05 | 2, 1.96 | 0.04, 0 | 0.76 | 0.71* |
| E1M5_213 | 37, 40 | 0.81, 0.9 | 0.57, 0.69 | 1.96, 1.74 | 0.5, 0.44 | 2, 2 | 0, 0 | 0.02 | 0.02 |
| E1M5_226 | 37, 40 | 0.97, 0.42 | 0.88, 0.25 | 1.28, 1.57 | 0.19, 0.36 | 2, 2 | 0, 0 | 0.36 | 0.37* |
| E1M5_240 | 37, 40 | 0.35, 0.27 | 0.19, 0.15 | 1.46, 1.36 | 0.32, 0.23 | 2, 2 | 0, 0 | 0.01 | 0 |
| E1M5_78 | 37, 40 | 0.3, 0 | 0.16, 0 | 1.37, 1 | 0.27, 0 | 2, 1 | 1, 0 | 0.17 | 0.21* |
| E1M5_88 | 37, 40 | 0.16, 0.53 | 0.09, 0.34 | 1.19, 1.7 | 0.16, 0.4 | 2, 2 | 0, 0 | 0.15 | 0.16* |

| | | | | | | | | | |
|---|---|---|---|---|---|---|---|---|---|
| E1M7_111 | 36, 38 | 0.7, 0.95 | 0.45, 0.81 | 1.96, 1.45 | 0.5, 0.28 | 2, 2 | 0, 0 | 0.11 | 0.1* |
| E1M7_117 | 36, 38 | 0.42, 0.27 | 0.24, 0.15 | 1.58, 1.34 | 0.37, 0.26 | 2, 2 | 0, 0 | 0.03 | 0.02 |
| E1M7_122 | 36, 38 | 0.67, 0.27 | 0.43, 0.15 | 1.92, 1.34 | 0.49, 0.25 | 2, 2 | 0, 0 | 0.16 | 0.18* |
| E1M7_153 | 36, 38 | 0.09, 0.85 | 0.05, 0.68 | 1.1, 1.66 | 0.09, 0.35 | 2, 2 | 0, 0 | 0.58 | 0.53* |
| E1M7_162 | 36, 38 | 0.03, 0.58 | 0.01, 0.36 | 1.03, 1.8 | 0.03, 0.46 | 1.99, 2 | 0, 0.01 | 0.36 | 0.33* |
| E1M7_163 | 36, 38 | 0.67, 0.63 | 0.45, 0.4 | 1.85, 1.85 | 0.47, 0.47 | 2, 2 | 0, 0 | 0 | -0.02 |
| E1M7_174 | 36, 38 | 0.86, 0.29 | 0.63, 0.16 | 1.87, 1.36 | 0.48, 0.28 | 2, 2 | 0, 0 | 0.33 | 0.34* |
| E1M7_177 | 36, 38 | 0.4, 0.45 | 0.23, 0.26 | 1.54, 1.62 | 0.35, 0.39 | 2, 2 | 0, 0 | 0 | -0.02 |
| E1M7_206 | 36, 38 | 0.06, 0.05 | 0.03, 0.03 | 1.06, 1.05 | 0.06, 0.05 | 1.99, 1.98 | 0.02, 0.01 | 0 | -0.02 |
| E1M7_211 | 36, 38 | 0.56, 0.82 | 0.34, 0.58 | 1.8, 1.9 | 0.45, 0.49 | 2, 2 | 0, 0 | 0.08 | 0.08* |
| E1M7_213 | 36, 38 | 0.83, 0.56 | 0.59, 0.34 | 1.93, 1.79 | 0.5, 0.46 | 2, 2 | 0, 0 | 0.09 | 0.08* |
| E1M7_246 | 36, 38 | 0.17, 0.51 | 0.09, 0.32 | 1.2, 1.66 | 0.17, 0.4 | 2, 2 | 0, 0 | 0.14 | 0.14* |
| E1M7_252 | 36, 38 | 0.39, 0.16 | 0.22, 0.09 | 1.53, 1.19 | 0.35, 0.16 | 2, 2 | 0, 0 | 0.07 | 0.07* |
| E1M7_266 | 36, 38 | 0.19, 1 | 0.1, 1 | 1.22, 1 | 0.19, 0 | 2, 1 | 1, 0 | 0.67 | 0.6* |
| E1M7_273 | 36, 38 | 0.22, 0.41 | 0.12, 0.24 | 1.26, 1.56 | 0.21, 0.34 | 2, 2 | 0, 0 | 0.03 | 0.02 |
| E1M7_275 | 36, 38 | 0.2, 0.71 | 0.11, 0.46 | 1.24, 1.94 | 0.19, 0.5 | 2, 2 | 0, 0 | 0.27 | 0.28* |

| | | | | | | | | | |
|---|---|---|---|---|---|---|---|---|---|
| E1M7_289 | 36, 38 | 0.03, 0 | 0.01, 0 | 1.03, 1 | 0.03, 0 | 1.99, 1 | 0.99, 0 | n/a | n/a |
| E1M7_346 | 36, 38 | 0.8, 0.19 | 0.56, 0.11 | 1.96, 1.25 | 0.5, 0.18 | 2, 2 | 0, 0 | 0.39 | 0.39* |
| E1M7_384 | 36, 38 | 0.03, 0.59 | 0.01, 0.38 | 1.03, 1.72 | 0.03, 0.43 | 1.99, 2 | 0, 0.01 | 0.39 | 0.36* |
| E1M7_453 | 36, 38 | 0.77, 0.06 | 0.53, 0.03 | 1.95, 1.06 | 0.5, 0.06 | 2, 1.98 | 0.02, 0 | 0.54 | 0.51* |
| E1M7_73 | 36, 38 | 0.09, 0 | 0.04, 0 | 1.09, 1 | 0.09, 0 | 2, 1 | 1, 0 | n/a | n/a |
| E1M7_79 | 36, 38 | 0.54, 0.71 | 0.33, 0.49 | 1.74, 1.75 | 0.42, 0.44 | 2, 2 | 0, 0 | 0.04 | 0.03 |
| E1M7_82 | 36, 38 | 0.06, 0.03 | 0.03, 0.01 | 1.06, 1.03 | 0.06, 0.03 | 1.99, 1.98 | 0.02, 0.01 | n/a | n/a |
| E1M7_83 | 36, 38 | 0.97, 0.22 | 0.88, 0.12 | 1.29, 1.27 | 0.19, 0.21 | 2, 2 | 0, 0 | 0.6 | 0.54* |
| E1M7_91 | 36, 38 | 0.03, 0.93 | 0.01, 0.85 | 1.03, 1.33 | 0.03, 0.17 | 1.99, 2 | 0, 0.01 | 0.8 | 0.67* |
| E4M7_105 | 40, 42 | 0.03, 0.05 | 0.01, 0.02 | 1.03, 1.05 | 0.03, 0.05 | 1.96, 1.95 | 0.05, 0.04 | n/a | n/a |
| E4M7_113 | 40, 42 | 0.55, 0.83 | 0.33, 0.59 | 1.78, 1.92 | 0.44, 0.5 | 2, 2 | 0, 0 | 0.09 | 0.15* |
| E4M7_139 | 40, 42 | 0.98, 0.88 | 0.89, 0.66 | 1.27, 1.81 | 0.18, 0.46 | 2, 2 | 0, 0 | 0.03 | 0.01 |
| E4M7_145 | 40, 42 | 0.1, 1 | 0.05, 1 | 1.11, 1 | 0.1, 0 | 2, 1 | 1, 0 | 0.82 | 0.86* |
| E4M7_146 | 40, 42 | 0.1, 0.83 | 0.05, 0.67 | 1.11, 1.66 | 0.1, 0.34 | 2, 2 | 0, 0 | 0.54 | 0.65* |
| E4M7_152 | 40, 42 | 0.55, 0 | 0.33, 0 | 1.79, 1 | 0.45, 0 | 2, 1 | 1, 0 | 0.38 | 0.52* |

| | | | | | | | | | |
|---|---|---|---|---|---|---|---|---|---|
| E4M7_179 | 40, 42 | 0.28, 0.6 | 0.15, 0.37 | 1.34, 1.83 | 0.26, 0.47 | 2, 2 | 0, 0 | 0.1 | 0.15* |
| E4M7_208 | 40, 42 | 0.2, 0.67 | 0.11, 0.43 | 1.24, 1.91 | 0.19, 0.49 | 2, 2 | 0, 0 | 0.22 | 0.32* |
| E4M7_263 | 40, 42 | 0.03, 0.69 | 0.01, 0.55 | 1.03, 1.49 | 0.03, 0.28 | 1.96, 2 | 0, 0.04 | 0.48 | 0.59* |
| E4M7_296 | 40, 42 | 0.95, 0.76 | 0.84, 0.54 | 1.38, 1.82 | 0.22, 0.46 | 2, 2 | 0, 0 | 0.07 | 0.08* |
| E4M7_301 | 40, 42 | 0.45, 0.48 | 0.26, 0.28 | 1.62, 1.67 | 0.39, 0.41 | 2, 2 | 0, 0 | 0 | -0.02 |
| E4M7_72 | 40, 42 | 0.43, 0.1 | 0.25, 0.05 | 1.58, 1.12 | 0.37, 0.09 | 2, 2 | 0, 0 | 0.14 | 0.22* |
| E4M7_80 | 40, 42 | 1, 1 | 1, 1 | 1, 1 | 0, 0 | 1, 1 | 0, 0 | n/a | n/a |
| E4M7_86 | 40, 42 | 0.2, 0.83 | 0.11, 0.61 | 1.24, 1.84 | 0.19, 0.47 | 2, 2 | 0, 0 | 0.4 | 0.53* |
| E4M7_88 | 40, 42 | 0.08, 0.6 | 0.04, 0.37 | 1.08, 1.84 | 0.08, 0.47 | 1.99, 2 | 0, 0.01 | 0.3 | 0.4* |
| E4M7_90 | 40, 42 | 0.63, 0.31 | 0.39, 0.17 | 1.89, 1.4 | 0.48, 0.28 | 2, 2 | 0, 0 | 0.1 | 0.15* |
| E5M3_104 | 36, 38 | 0.44, 0.77 | 0.26, 0.52 | 1.61, 1.98 | 0.38, 0.51 | 2, 2 | 0, 0 | 0.11 | 0.11* |
| E5M3_121 | 36, 38 | 0.44, 0.24 | 0.25, 0.13 | 1.61, 1.29 | 0.39, 0.23 | 2, 2 | 0, 0 | 0.05 | 0.04 |
| E5M3_122 | 36, 38 | 0.22, 0.87 | 0.12, 0.64 | 1.26, 1.84 | 0.21, 0.47 | 2, 2 | 0, 0 | 0.42 | 0.42* |
| E5M3_189 | 36, 38 | 0, 0 | 0, 0 | 1, 1 | 0, 0 | 1, 1 | 0, 0 | n/a | n/a |
| E5M3_214 | 36, 38 | 0.31, 0.97 | 0.17, 0.9 | 1.39, 1.23 | 0.29, 0.14 | 2, 2 | 0, 0 | 0.48 | 0.47* |
| E5M3_219 | 36, 38 | 0.03, 0.03 | 0.01, 0.01 | 1.03, 1.03 | 0.03, 0.03 | 1.99, 1.98 | 0.02, 0.01 | n/a | n/a |

| | | | | | | | | | |
|---|---|---|---|---|---|---|---|---|---|
| E5M3_270 | 36, 38 | 0.75, 0.08 | 0.51, 0.04 | 1.94, 1.09 | 0.5, 0.08 | 2, 2 | 0, 0 | 0.46 | 0.45* |
| E5M3_295 | 36, 38 | 0, 0.31 | 0, 0.17 | 1, 1.41 | 0, 0.28 | 1, 2 | 0, 1 | 0.19 | 0.13* |
| E5M3_302 | 36, 38 | 0.67, 0.19 | 0.42, 0.11 | 1.95, 1.24 | 0.5, 0.18 | 2, 2 | 0, 0 | 0.24 | 0.25* |
| E5M3_405 | 36, 38 | 0.94, 0.31 | 0.83, 0.18 | 1.4, 1.42 | 0.23, 0.26 | 2, 2 | 0, 0 | 0.45 | 0.43* |
| E5M3_414 | 36, 38 | 0, 0 | 0, 0 | 1, 1 | 0, 0 | 1, 1 | 0, 0 | n/a | n/a |
| E5M3_64 | 35, 38 | 0.06, 1 | 0.03, 1 | 1.06, 1 | 0.06, 0 | 1.99, 1 | 1, 0.01 | 0.89 | 0.71* |
| E5M3_65 | 35, 38 | 1, 0.14 | 1, 0.07 | 1, 1.16 | 0, 0.14 | 1, 2 | 0, 1 | 0.77 | 0.63* |
| E5M3_75 | 36, 38 | 0, 0.02 | 0, 0.01 | 1, 1.03 | 0, 0.02 | 1, 1.98 | 0, 0.98 | n/a | n/a |
| E5M3_83 | 36, 38 | 0.08, 0.78 | 0.04, 0.55 | 1.09, 1.87 | 0.08, 0.48 | 2, 2 | 0, 0 | 0.51 | 0.48* |
| E5M3_90 | 36, 38 | 0.03, 0.13 | 0.01, 0.07 | 1.03, 1.15 | 0.03, 0.13 | 1.99, 2 | 0, 0.01 | 0.04 | 0 |
| E5M3_96 | 36, 38 | 0.06, 0.03 | 0.03, 0.01 | 1.06, 1.03 | 0.06, 0.03 | 1.99, 1.98 | 0.02, 0.01 | 0.01 | -0.01 |
| E5M6_103 | 40, 42 | 0.45, 0.52 | 0.26, 0.31 | 1.62, 1.74 | 0.39, 0.44 | 2, 2 | 0, 0 | 0.01 | -0.01 |
| E5M6_123 | 40, 42 | 0.1, 0 | 0.05, 0 | 1.12, 1 | 0.1, 0 | 2, 1 | 1, 0 | n/a | n/a |
| E5M6_136 | 40, 42 | 0.98, 0.38 | 0.89, 0.22 | 1.27, 1.51 | 0.18, 0.34 | 2, 2 | 0, 0 | 0.4 | 0.51* |
| E5M6_187 | 40, 42 | 0, 0 | 0, 0 | 1, 1 | 0, 0 | 1, 1 | 0, 0 | n/a | n/a |
| E5M6_194 | 40, 42 | 1, 0.93 | 1, 0.78 | 1, 1.51 | 0, 0.3 | 1, 2 | 0, 1 | n/a | n/a |

| | | | | | | | | | |
|---|---|---|---|---|---|---|---|---|---|
| E5M6_208 | 40, 42 | 0.68, 0.38 | 0.43, 0.22 | 1.96, 1.51 | 0.5, 0.34 | 2, 2 | 0, 0 | 0.09 | 0.13* |
| E5M6_233 | 40, 42 | 0.23, 0 | 0.12, 0 | 1.27, 1 | 0.22, 0 | 2, 1 | 1, 0 | 0.13 | 0.2* |
| E5M6_324 | 40, 42 | 0.78, 0 | 0.53, 0 | 1.94, 1 | 0.5, 0 | 2, 1 | 1, 0 | 0.63 | 0.73* |
| E5M6_396 | 40, 42 | 0.2, 1 | 0.11, 1 | 1.24, 1 | 0.19, 0 | 2, 1 | 1, 0 | 0.67 | 0.76* |
| E5M6_397 | 40, 42 | 0.63, 0.9 | 0.41, 0.76 | 1.8, 1.54 | 0.45, 0.31 | 2, 2 | 0, 0 | 0.11 | 0.18* |
| E5M6_401 | 40, 42 | 0.98, 0 | 0.89, 0 | 1.27, 1 | 0.18, 0 | 2, 1 | 1, 0 | 0.95 | 0.93* |
| E5M6_453 | 40, 42 | 0.05, 0 | 0.03, 0 | 1.05, 1 | 0.05, 0 | 1.96, 1 | 0.96, 0 | n/a | n/a |
| E5M6_65 | 40, 42 | 0.7, 0 | 0.46, 0 | 1.92, 1 | 0.49, 0 | 2, 1 | 1, 0 | 0.54 | 0.66* |
| E5M6_75 | 40, 42 | 0.25, 1 | 0.14, 1 | 1.32, 1 | 0.23, 0 | 2, 1 | 1, 0 | 0.6 | 0.71* |
| E5M6_90 | 40, 42 | 0.1, 0.64 | 0.05, 0.44 | 1.11, 1.69 | 0.1, 0.42 | 2, 2 | 0, 0 | 0.32 | 0.42* |
| E5M6_93 | 40, 42 | 0.98, 0 | 0.89, 0 | 1.27, 1 | 0.18, 0 | 2, 1 | 1, 0 | 0.95 | 0.93* |
| E5M6_96 | 40, 42 | 0.15, 0.88 | 0.08, 0.73 | 1.17, 1.54 | 0.15, 0.31 | 2, 2 | 0, 0 | 0.53 | 0.65* |
| E5M6_98 | 40, 42 | 0.7, 0 | 0.45, 0 | 1.97, 1 | 0.5, 0 | 2, 1 | 1, 0 | 0.54 | 0.66* |
| E8M5_101 | 37, 40 | 0.11, 0.11 | 0.06, 0.06 | 1.12, 1.13 | 0.1, 0.1 | 2, 2 | 0, 0 | 0 | -0.01 |
| E8M5_106 | 37, 40 | 0.59, 0.89 | 0.37, 0.74 | 1.81, 1.55 | 0.45, 0.31 | 2, 2 | 0, 0 | 0.12 | 0.16* |
| E8M5_153 | 37, 40 | 0.56, 1 | 0.35, 1 | 1.76, 1 | 0.43, 0 | 2, 1 | 1, 0 | 0.28 | 0.33* |
| E8M5_154 | 37, 40 | 0.81, 0.03 | 0.6, 0.01 | 1.77, 1.03 | 0.44, 0.03 | 2, 1.96 | 0.04, 0 | 0.63 | 0.64* |

| | | | | | | | | | |
|---|---|---|---|---|---|---|---|---|---|
| E8M5_157 | 37, 40 | 0.46, 0.12 | 0.26, 0.07 | 1.64, 1.15 | 0.4, 0.12 | 2, 2 | 0, 0 | 0.14 | 0.18* |
| E8M5_168 | 37, 40 | 0.78, 1 | 0.54, 1 | 1.96, 1 | 0.5, 0 | 2, 1 | 1, 0 | 0.12 | 0.14* |
| E8M5_186 | 37, 40 | 0.08, 0.95 | 0.04, 0.81 | 1.09, 1.45 | 0.08, 0.28 | 2, 2 | 0, 0 | 0.76 | 0.71* |
| E8M5_195 | 37, 40 | 0.08, 0.98 | 0.04, 0.91 | 1.09, 1.21 | 0.08, 0.14 | 2, 2 | 0, 0 | 0.8 | 0.74* |
| E8M5_196 | 37, 40 | 0.03, 0.45 | 0.01, 0.26 | 1.03, 1.62 | 0.03, 0.4 | 1.98, 2 | 0, 0.02 | 0.25 | 0.24* |
| E8M5_205 | 37, 40 | 0, 0.02 | 0, 0.01 | 1, 1.03 | 0, 0.02 | 1, 1.96 | 0, 0.96 | n/a | n/a |
| E8M5_232 | 37, 40 | 0.35, 0.06 | 0.19, 0.03 | 1.46, 1.06 | 0.32, 0.06 | 2, 1.96 | 0.04, 0 | 0.14 | 0.18* |
| E8M5_271 | 37, 40 | 1, 0.98 | 1, 0.91 | 1, 1.21 | 0, 0.14 | 1, 2 | 0, 1 | n/a | n/a |
| E8M5_293 | 37, 40 | 0.11, 0.36 | 0.06, 0.22 | 1.12, 1.52 | 0.11, 0.3 | 2, 2 | 0, 0 | 0.08 | 0.07* |
| E8M7_102 | 36, 38 | 0.5, 0.03 | 0.29, 0.01 | 1.7, 1.03 | 0.42, 0.03 | 2, 1.98 | 0.02, 0 | 0.29 | 0.29* |
| E8M7_104 | 36, 38 | 0.31, 0.79 | 0.17, 0.54 | 1.39, 1.97 | 0.29, 0.51 | 2, 2 | 0, 0 | 0.24 | 0.25* |
| E8M7_119 | 36, 38 | 0.66, 0.77 | 0.42, 0.54 | 1.92, 1.87 | 0.49, 0.48 | 2, 2 | 0, 0 | 0.01 | 0 |
| E8M7_139 | 36, 38 | 0, 0 | 0, 0 | 1, 1 | 0, 0 | 1, 1 | 0, 0 | n/a | n/a |
| E8M7_200 | 36, 38 | 0, 0.43 | 0, 0.26 | 1, 1.55 | 0, 0.35 | 1, 2 | 0, 1 | 0.27 | 0.22* |
| E8M7_223 | 36, 38 | 0.27, 0.34 | 0.15, 0.19 | 1.34, 1.44 | 0.25, 0.32 | 2, 2 | 0, 0 | 0 | -0.02 |
| E8M7_226 | 36, 38 | 0.87, 1 | 0.65, 1 | 1.78, 1 | 0.44, 0 | 2, 1 | 1, 0 | 0.07 | 0.05 |
| E8M7_283 | 36, 38 | 0.28, 0 | 0.16, 0 | 1.37, 1 | 0.26, 0 | 2, 1 | 1, 0 | 0.16 | 0.14* |

| | | | | | | | | | |
|---|---|---|---|---|---|---|---|---|---|
| E8M7_300 | 36, 38 | 0, 0 | 0, 0 | 1, 1 | 0, 0 | 1, 1 | 0, 0 | n/a | n/a |
| E8M7_57 | 36, 38 | 1, 1 | 1, 1 | 1, 1 | 0, 0 | 1, 1 | 0, 0 | n/a | n/a |
| E8M7_62 | 36, 38 | 0.22, 0.35 | 0.12, 0.21 | 1.26, 1.46 | 0.21, 0.28 | 2, 2 | 0, 0 | 0.03 | 0.01 |
| E8M7_78 | 36, 38 | 0, 0 | 0, 0 | 1, 1 | 0, 0 | 1, 1 | 0, 0 | n/a | n/a |
| E8M7_79 | 36, 38 | 0.09, 0 | 0.04, 0 | 1.09, 1 | 0.09, 0 | 2, 1 | 1, 0 | n/a | n/a |
| E8M7_80 | 36, 38 | 0.89, 0.16 | 0.67, 0.08 | 1.8, 1.18 | 0.46, 0.16 | 2, 2 | 0, 0 | 0.54 | 0.5* |
| E8M7_94 | 36, 38 | 0.06, 0.65 | 0.03, 0.42 | 1.06, 1.89 | 0.06, 0.49 | 1.99, 2 | 0, 0.01 | 0.4 | 0.38* |
| E8M7_95 | 36, 38 | 0.97, 0.94 | 0.89, 0.86 | 1.27, 1.31 | 0.18, 0.17 | 2, 2 | 0, 0 | 0 | -0.02 |
| E8M7_98 | 36, 38 | 0.09, 0.33 | 0.05, 0.18 | 1.1, 1.43 | 0.09, 0.3 | 2, 2 | 0, 0 | 0.08 | 0.06* |
| EC733_260 | 35, 39 | 0.97, 0.03 | 0.88, 0.01 | 1.28, 1.03 | 0.19, 0.03 | 2, 1.97 | 0.03, 0 | 0.89 | 0.73* |
| EC733_263 | 35, 39 | 0.86, 0.95 | 0.62, 0.87 | 1.88, 1.3 | 0.48, 0.17 | 2, 2 | 0, 0 | 0.02 | 0.04 |
| EC733_264 | 35, 39 | 0.34, 0.67 | 0.19, 0.43 | 1.44, 1.93 | 0.31, 0.5 | 2, 2 | 0, 0 | 0.1 | 0.12* |
| EC733_265 | 35, 39 | 0.06, 0.8 | 0.03, 0.67 | 1.06, 1.43 | 0.06, 0.27 | 1.99, 2 | 0, 0.01 | 0.56 | 0.51* |
| EC733_270 | 35, 39 | 0.03, 0 | 0.01, 0 | 1.03, 1 | 0.03, 0 | 1.99, 1 | 0.99, 0 | n/a | n/a |
| EC978_254 | 42, 38 | 0, 0 | 0, 0 | 1, 1 | 0, 0 | 1, 1 | 0, 0 | n/a | n/a |
| EC978_255 | 42, 38 | 0, 0 | 0, 0 | 1, 1 | 0, 0 | 1, 1 | 0, 0 | n/a | n/a |
| EC978_256 | 42, 38 | 0.14, 0 | 0.07, 0 | 1.16, 1 | 0.14, 0 | 2, 1 | 1, 0 | 0.08 | 0.05 |

| | | | | | | | | | |
|---|---|---|---|---|---|---|---|---|---|
| EC978_257 | 42, 38 | 0.79, 0.18 | 0.54, 0.1 | 1.94, 1.21 | 0.5, 0.18 | 2, 2 | 0, 0 | 0.36 | 0.46* |
| EC978_260 | 42, 38 | 0.48, 0 | 0.29, 0 | 1.65, 1 | 0.38, 0 | 2, 1 | 1, 0 | 0.31 | 0.35* |
| EC978_262 | 42, 38 | 0.07, 0 | 0.04, 0 | 1.08, 1 | 0.07, 0 | 1.99, 1 | 0.99, 0 | n/a | n/a |
| ES72_261 | 40, 32 | 0.58, 0 | 0.35, 0 | 1.82, 1 | 0.46, 0 | 2, 1 | 1, 0 | 0.4 | 0.32* |
| ES72_264 | 40, 32 | 0.73, 0.56 | 0.49, 0.34 | 1.89, 1.81 | 0.48, 0.47 | 2, 2 | 0, 0 | 0.03 | 0.07* |
| ES72_267 | 40, 32 | 0.73, 0.36 | 0.49, 0.21 | 1.89, 1.49 | 0.48, 0.33 | 2, 2 | 0, 0 | 0.12 | 0.16* |
| ES72_270 | 40, 32 | 0.45, 0.94 | 0.26, 0.8 | 1.62, 1.47 | 0.39, 0.29 | 2, 2 | 0, 0 | 0.28 | 0.24* |
| ES74B_161 | 42, 42 | 0, 0 | 0, 0 | 1, 1 | 0, 0 | 1, 1 | 0, 0 | n/a | n/a |
| ES74B_164 | 42, 42 | 1, 1 | 1, 1 | 1, 1 | 0, 0 | 1, 1 | 0, 0 | n/a | n/a |
| ES74B_166 | 42, 42 | 0.24, 0.55 | 0.13, 0.33 | 1.29, 1.78 | 0.22, 0.45 | 2, 2 | 0, 0 | 0.1 | 0.16* |
| ES74B_167 | 42, 42 | 0.24, 0 | 0.13, 0 | 1.32, 1 | 0.21, 0 | 2, 1 | 1, 0 | 0.14 | 0.22* |
| ES74B_168 | 42, 42 | 0, 0 | 0, 0 | 1, 1 | 0, 0 | 1, 1 | 0, 0 | n/a | n/a |
| ES74B_169 | 42, 42 | 0, 0.02 | 0, 0.01 | 1, 1.03 | 0, 0.02 | 1, 1.95 | 0, 0.95 | n/a | n/a |
| ES74B_170 | 42, 42 | 0.17, 0.29 | 0.09, 0.16 | 1.19, 1.36 | 0.16, 0.27 | 2, 2 | 0, 0 | 0.02 | 0.02 |
| All AFLP loci | 37.05, 39.25 | 0.41, 0.43 | 0.3, 0.32 | 1.37, 1.37 | 0.23, 0.23 | 1.85, 1.76 | 0.18, 0.09 | 0.28 | 0.28* |
| All Dom loci | 40.05, | 0.36, 0.29 | 0.26, 0.22 | 1.34, 1.22 | 0.21, 0.14 | 1.73, 1.5 | 0.27, | 0.24 | 0.25* |

| | | | | | | | | | |
|---|---|---|---|---|---|---|---|---|---|
| | 38.41 | | | | | | 0.04 | | |
| All loci | 37.43, 39.15 | 0.41, 0.42 | 0.29, 0.31 | 1.37, 1.35 | 0.23, 0.21 | 1.83, 1.72 | 0.19, 0.08 | 0.28 | 0.28* |

**Legend to Table S7.** Where summary statistics are presented for both species, the species order is: *S. aethnensis*, *S. chrysanthemifolius*. Values were averaged across population samples within each species. *N* is total number of genotyped individuals. *Band Freq.* is observed band present frequency. *p* is expected band present allele frequency assuming Hardy-Weinberg equilibrium, *p* = 1/squared band freq. $N_e$ is effective number of alleles calculated as 1/sum($p^2$). *UHe* is unbiased expected heterozygosity calculated as (2*N*/(2*N*-1))*(1 – sum($p^2$)). *Ar* and *pAr* are allelic richness and private allelic richness after rarefaction to minimum common sample size of 64 alleles per species. $F_{ST}$ is allelic differentiation calculated as between-species variance/total variance using allele frequencies estimated under complete outcrossing. $Phi_{PT}$ is genotypic differentiation calculated as between-species variance/total variance. Asterisks after $Phi_{PT}$ values indicate significantly differentiated loci following 1000 data permutations.

**Table S8.** Summary population genetic statistics for codominantly scored molecular genetic markers from *S. aethnensis* and *S. chrysanthemifolius* samples.

| Locus | *N* | $N_e$ | *MAF* | $UH_e$ | *Ar* | *pAr* | $F_{IS}$ | $F_{ST}$ | $Phi_{PT}$ |
|---|---|---|---|---|---|---|---|---|---|
| A26 | 41, 40 | 2.08, 2 | 0.44, 0.31 | 0.53, 0.45 | 2.9, 3.96 | 0.04, 1.09 | 0.18, -0.1 | 0.26* | 0.49* |
| C19 | 41, 40 | 1.05, 1.7 | 0.02, 0.31 | 0.05, 0.41 | 1.79, 2 | 0, 0.21 | -0.03, -0.08 | 0.15* | 0.31* |
| EC1026 | 41, 33 | 1.98, 1.57 | 0.46, 0.27 | 0.51, 0.32 | 2, 2 | 0, 0 | 0.31, 1 | 0.04 | 0.08* |
| EC1307 | 40, 38 | 1, 1 | 0, 0 | 0, 0 | 1, 1 | 0, 0 | n/a, n/a | n/a | -0.01 |
| EC1418 | 35, 39 | 1.03, 1.35 | 0.01, 0.15 | 0.03, 0.26 | 1.63, 2 | 0, 0.37 | -0.03, -0.19 | 0.06* | 0.03 |
| EC1551 | 42, 41 | 1.65, 1.53 | 0.27, 0.22 | 0.39, 0.33 | 2, 2 | 0, 0 | 0.07, 0.04 | 0 | -0.01 |
| EC1566 | 35, 38 | 3.4, 2.36 | 0.59, 0.43 | 0.73, 0.57 | 5.73, 4.54 | 1.33, 0.14 | -0.02, 0.06 | 0.06* | 0.12* |
| EC1687 | 35, 39 | 1.68, 1.9 | 0.29, 0.42 | 0.41, 0.49 | 2, 2 | 0, 0 | 0.13, -0.62 | 0.02* | 0.03 |
| EC1688 | 35, 22 | 2.92, 3.08 | 0.49, 0.55 | 0.66, 0.71 | 5.73, 5 | 1.87, 1.14 | -0.1, -0.17 | 0.09* | 0.19* |
| EC174 | 41, 40 | 2.73, 1.9 | 0.51, 0.26 | 0.65, 0.39 | 4.49, 4.52 | 0.46, 0.49 | -0.19, -0.01 | 0.15* | 0.35* |
| EC232 | 39, 40 | 1.03, 1.75 | 0.01, 0.31 | 0.03, 0.44 | 1.56, 2 | 0, 0.44 | -0.03, -0.1 | 0.16* | 0.26* |
| EC371 | 37, 37 | 1.72, 1.46 | 0.3, 0.19 | 0.43, 0.31 | 2, 2 | 0, 0 | -0.03, 0.02 | 0.02 | 0 |

| | | | | | | | | | |
|---|---|---|---|---|---|---|---|---|---|
| EC402 | 39, 28 | 3.32, 1 | 0.64, 0 | 0.72, 0 | 3.99, 1 | 3, 0.01 | 0.16, n/a | 0.45* | 0.41* |
| EC63 | 41, 39 | 1, 1 | 0, 0 | 0, 0 | 1, 1 | 0, 0 | n/a, n/a | n/a | 0 |
| EC77 | 42, 32 | 1.76, 1.4 | 0.27, 0.17 | 0.44, 0.28 | 3, 2.91 | 0.09, 0 | -0.27, -0.2 | 0.38* | 0.56* |
| EC811 | 36, 36 | 1.03, 1.85 | 0.01, 0.4 | 0.03, 0.47 | 1.61, 2 | 0, 0.39 | -0.03, 0.02 | 0.4* | 0.41* |
| EC859 | 42, 40 | 2.94, 2.86 | 0.49, 0.59 | 0.68, 0.67 | 5.94, 4.55 | 2.97, 1.58 | 0.06, 0.14 | 0.17* | 0.38* |
| Ray2aB | 35, 38 | 1.06, 1.35 | 0.03, 0.16 | 0.06, 0.25 | 1.87, 2 | 0, 0.13 | -0.06, -0.04 | 0.67* | 0.63* |
| SSP | 41, 42 | 1.11, 1.44 | 0.05, 0.17 | 0.09, 0.26 | 1.96, 2.95 | 0, 0.99 | -0.11, -0.09 | 0.03* | 0.05 |
| EC1019 | 41, 38 | 1.02, 1.05 | 0.01, 0.03 | 0.02, 0.05 | 1.54, 1.83 | 0.09, 0.38 | -0.02, -0.08 | 0 | 0.02 |
| EC1123 | 40, 36 | 1.08, 1.49 | 0.04, 0.21 | 0.07, 0.34 | 1.91, 2 | 0, 0.09 | -0.04, 0.11 | 0.59* | 0.65* |
| EC1470 | 39, 39 | 1.46, 1.54 | 0.19, 0.23 | 0.32, 0.34 | 2.56, 2 | 1, 0.44 | -0.07, 0.04 | 0.06* | 0.09* |
| EC1496B | 40, 35 | 1.16, 1.19 | 0.08, 0.09 | 0.14, 0.15 | 1.99, 2 | 0, 0.01 | -0.08, 0.1 | 0.7* | 0.7* |
| EC258 | 41, 33 | 1.84, 1.65 | 0.35, 0.27 | 0.47, 0.39 | 2, 2 | 0, 0 | 0, -0.05 | 0.01 | 0.05* |
| EC290 | 41, 39 | 1.96, 1.11 | 0.33, 0.05 | 0.5, 0.1 | 3, 1.97 | 1.03, 0 | 0.41, 1 | 0.46* | 0.59* |
| EC296B | 41, 38 | 1.05, 1.51 | 0.02, 0.2 | 0.05, 0.32 | 1.78, 3.8 | 0.2, 2.21 | -0.05, 0 | 0.67* | 0.8* |
| EC298 | 41, 40 | 1, 1 | 0, 0 | 0, 0 | 1, 1 | 0, 0 | n/a, n/a | n/a | 0 |
| EC370 | 41, 34 | 2.41, 1.8 | 0.5, 0.34 | 0.6, 0.46 | 3, 2 | 1, 0 | -0.05, 0.28 | 0.1* | 0.19* |
| EC464 | 38, 33 | 4.36, 2.54 | 0.67, 0.48 | 0.78, 0.62 | 6.73, 3.99 | 4.01, 1.27 | 0.22, -0.18 | 0.15* | 0.27* |

| | | | | | | | | | |
|---|---|---|---|---|---|---|---|---|---|
| EC482 | 35, 35 | 1.1, 1.91 | 0.04, 0.41 | 0.08, 0.5 | 1.95, 2.86 | 0, 0.91 | -0.1, 0.13 | 0.34* | 0.35* |
| EC512 | 37, 37 | 2.08, 2.21 | 0.42, 0.51 | 0.53, 0.56 | 2.97, 3 | 0, 0.03 | 0.17, 0.01 | 0.17* | 0.28* |
| EC606 | 42, 39 | 1, 1 | 0, 0 | 0, 0 | 1, 1 | 0, 0 | n/a, n/a | n/a | 0.05 |
| EC688 | 40, 31 | 1.88, 1.49 | 0.39, 0.23 | 0.48, 0.3 | 2, 2 | 0, 0 | -0.11, -0.02 | 0.15* | 0.25* |
| EC74 | 41, 33 | 3.39, 1.36 | 0.55, 0.15 | 0.71, 0.2 | 5.78, 2.67 | 3.25, 0.14 | 0.02, -0.03 | 0.28* | 0.39* |
| EC904 | 40, 38 | 1.41, 1.84 | 0.16, 0.36 | 0.3, 0.47 | 3.82, 3.92 | 1.07, 1.16 | -0.13, 0.14 | 0.4* | 0.59* |
| ES1 | 37, 35 | 2.34, 2.11 | 0.45, 0.51 | 0.59, 0.54 | 3.93, 2.99 | 1.99, 1.06 | 0.05, -0.06 | 0.11* | 0.18* |
| ES18 | 42, 40 | 1, 1 | 0, 0 | 0, 0 | 1, 1 | 0, 0 | n/a, n/a | n/a | 0.02 |
| ES19 | 34, 39 | 3.23, 2.02 | 0.59, 0.38 | 0.71, 0.5 | 4.65, 3 | 3, 1.35 | 0.05, -0.15 | 0.22* | 0.38* |
| ES2 | 34, 24 | 2.53, 3.36 | 0.47, 0.71 | 0.62, 0.74 | 5.69, 7.8 | 1.97, 4.08 | -0.27, 0.05 | 0.15* | 0.25* |
| ES20 | 35, 36 | 2.75, 1.12 | 0.54, 0.06 | 0.66, 0.1 | 3.87, 2.7 | 1.16, 0 | -0.03, -0.07 | 0.38* | 0.47* |
| ES25 | 41, 37 | 1.19, 1 | 0.09, 0 | 0.16, 0 | 2, 1 | 1, 0 | -0.09, n/a | 0.04* | 0.02 |
| ES29 | 42, 42 | 1.07, 1.74 | 0.04, 0.45 | 0.07, 0.44 | 1.9, 2 | 0, 0.1 | -0.04, 0.55 | 0.24* | 0.43* |
| ES36 | 40, 42 | 1.75, 1.13 | 0.31, 0.06 | 0.44, 0.12 | 2, 2.48 | 0.52, 1 | -0.21, -0.06 | 0.56* | 0.82* |
| ES4 | 40, 40 | 4.52, 3.89 | 0.6, 0.56 | 0.79, 0.74 | 11.12, 8.58 | 4.82, 2.28 | 0, 0.16 | 0.07* | 0.17* |
| ES40 | 40, 39 | 2.56, 1.19 | 0.45, 0.08 | 0.62, 0.14 | 4, 2.78 | 1.22, 0 | 0.06, 0.01 | 0.39* | 0.59* |
| ES43 | 40, 39 | 1.49, 2.25 | 0.21, 0.45 | 0.3, 0.56 | 2, 3 | 0, 1 | 0.03, 0.27 | 0.2* | 0.34* |

| | | | | | | | | | |
|---|---|---|---|---|---|---|---|---|---|
| ES45 | 42, 42 | 2.32, 3.97 | 0.38, 0.69 | 0.57, 0.77 | 6.07, 5 | 1.78, 0.71 | 0.07, -0.06 | 0.12* | 0.32* |
| ES47 | 40, 25 | 1.57, 1.17 | 0.24, 0.08 | 0.37, 0.14 | 2, 2 | 0, 0 | 0.11, 0.44 | 0.05* | 0.2* |
| ES56 | 40, 41 | 1.26, 3.62 | 0.11, 0.73 | 0.21, 0.75 | 2.95, 4 | 0.96, 2.02 | 0.18, 0.16 | 0.23* | 0.43* |
| ES57 | 41, 42 | 2.68, 2.65 | 0.49, 0.67 | 0.64, 0.64 | 4.52, 4 | 1, 0.48 | -0.06, 0.24 | 0.06* | 0.17* |
| ES58 | 40, 27 | 1.34, 1.67 | 0.15, 0.3 | 0.26, 0.41 | 2, 2 | 0, 0 | 0.02, 0.46 | 0.31* | 0.41* |
| ES63 | 41, 41 | 1, 1 | 0, 0 | 0, 0 | 1, 1 | 0, 0 | n/a, n/a | n/a | -0.02 |
| ES64 | 41, 35 | 1, 1 | 0, 0 | 0, 0 | 1, 1 | 0, 0 | n/a, n/a | n/a | 0.09 |
| ES73 | 42, 41 | 1.66, 1.78 | 0.25, 0.33 | 0.41, 0.45 | 3, 2 | 1, 0 | 0.16, 0.16 | 0.25* | 0.49* |
| ES76 | 35, 36 | 1.44, 1 | 0.19, 0 | 0.31, 0 | 2, 1 | 1, 0 | -0.07, n/a | 0.1* | 0.06* |
| ES82 | 42, 42 | 1, 1.51 | 0, 0.2 | 0, 0.34 | 1, 2.78 | 0, 1.78 | n/a, -0.12 | 0.1* | 0.31* |
| ES83B | 41, 35 | 3, 2.01 | 0.59, 0.34 | 0.67, 0.51 | 5.47, 4.49 | 1.49, 0.51 | 0.26, -0.17 | 0.22* | 0.38* |
| ES85 | 42, 41 | 2.02, 1.4 | 0.33, 0.17 | 0.51, 0.29 | 3.78, 2.54 | 1.24, 0 | 0, 0.18 | 0.02* | 0.05 |
| ES87 | 36, 34 | 1.45, 1.76 | 0.18, 0.5 | 0.32, 0.45 | 2.98, 2 | 1, 0.02 | 0.74, 0.3 | 0.16* | 0.18* |
| ES88 | 38, 40 | 2.25, 2.32 | 0.38, 0.54 | 0.57, 0.59 | 4.91, 3.55 | 1.45, 0.09 | 0.09, -0.03 | 0.1* | 0.21* |
| ES9 | 40, 38 | 2.11, 2.24 | 0.38, 0.36 | 0.53, 0.54 | 4.08, 3.99 | 1, 0.9 | -0.03, -0.06 | 0.19* | 0.37* |
| ES91 | 40, 33 | 3.03, 1.67 | 0.61, 0.27 | 0.69, 0.42 | 4.35, 2.67 | 1.98, 0.3 | 0.1, 0.1 | 0.12* | 0.18* |
| NES1B | 39, 35 | 3.15, 2.21 | 0.55, 0.49 | 0.7, 0.54 | 5.13, 5.21 | 2.77, 2.85 | 0.29, -0.19 | 0.19* | 0.32* |

| | | | | | | | | | |
|---|---|---|---|---|---|---|---|---|---|
| MSC5a | 40, 41 | 2.16, 2.11 | 0.49, 0.38 | 0.55, 0.54 | 2.96, 3.9 | 0.1, 1.04 | 0.06, 0.3 | 0.25* | 0.47* |
| MSC5b | 40, 40 | 2.89, 4.54 | 0.59, 0.78 | 0.67, 0.81 | 5.15, 8.48 | 1.26, 4.59 | 0, 0.29 | 0.12* | 0.27* |
| MSS7 | 40, 32 | 2.52, 1.17 | 0.48, 0.08 | 0.61, 0.15 | 3, 2 | 1, 0 | -0.27, -0.08 | 0.3* | 0.39* |
| S10 | 38, 38 | 3.05, 2.45 | 0.61, 0.42 | 0.69, 0.57 | 4.55, 3.99 | 0.99, 0.43 | -0.02, 0.09 | 0.02* | 0.04* |
| S20 | 39, 37 | 2.33, 3.9 | 0.36, 0.74 | 0.57, 0.72 | 7.21, 8.35 | 1.27, 2.41 | -0.13, -0.14 | 0.14* | 0.31* |
| S26 | 39, 42 | 2.95, 2.23 | 0.56, 0.45 | 0.67, 0.54 | 4.8, 3.52 | 1.48, 0.19 | 0.13, 0.01 | 0.12* | 0.28* |
| S29 | 38, 35 | 1.54, 1.64 | 0.22, 0.27 | 0.34, 0.39 | 2.83, 2 | 1, 0.17 | 0.08, 0.09 | 0.34* | 0.43* |
| V44 | 42, 42 | 3.05, 3.18 | 0.6, 0.64 | 0.69, 0.7 | 4.55, 7.58 | 0.78, 3.8 | 0.05, -0.1 | 0.02* | 0.05* |
| V45 | 39, 36 | 5.33, 2.91 | 0.71, 0.4 | 0.83, 0.58 | 8.52, 8.37 | 1.54, 1.4 | 0.05, -0.08 | 0.04* | 0.07* |
| ESTindels | 38.84, 36.95 | 1.82, 1.71 | 0.26, 0.26 | 0.34, 0.35 | 2.75, 2.6 | 0.51, 0.37 | 0, -0.02 | 0.18* | 0.23* |
| ESTSSRs | 39.61, 36.8 | 1.93, 1.78 | 0.28, 0.28 | 0.38, 0.35 | 3.21, 2.83 | 1, 0.62 | 0.04, 0.1 | 0.22* | 0.3* |
| SSRs | 39.44, 38.11 | 2.87, 2.68 | 0.51, 0.46 | 0.62, 0.56 | 4.84, 5.35 | 1.05, 1.56 | -0.01, 0.04 | 0.15* | 0.26* |
| All loci | 39.39, 37 | 2.02, 1.88 | 0.3, 0.3 | 0.4, 0.38 | 3.29, 3.09 | 0.88, 0.67 | 0.02, 0.06 | 0.2* | 0.27* |

**Legend to Table S8.** Where summary statistics are presented for both species, the species order is; *S. aethnensis*, *S. chrysanthemifolius*. Values were averaged across population samples within each species. See Table S6 for a description of most of the presented statistics except for the following additional codominant marker-specific statistics. *MAF* is the sum frequency of all minor frequency alleles calculated as 1 – most frequent allele. $F_{IS}$ is the fixation index calculated as 1- (the number of observed heterozygotes/ *UHe*). $F_{ST}$ is allelic differentiation and is calculated as between-species variance/total variance using observed allele frequencies for codominant markers.

**Table S9.** Expressed sequence tag (EST) loci showing evidence for divergent or convergent selection between *S. aethnensis* and *S. chrysanthemifolius*.

| Analysis | Marker | Marker type | Source EST or sequence | Linkage group; position | Selection type | $Log_{10}$ Bayes Factor | Est. $F_{ST}$ | Best match gene; expect value | *Arabidopsis thaliana* best match gene; expect value |
|---|---|---|---|---|---|---|---|---|---|
| Both | EC296B | EST SSR | multiple | 1; 0 | Divergent | >5.00; 1.53 | 0.59; 0.50 | AF493943.1; Artemisia vulgaris major pollen allergen Art v 1 precursor; 6e-11 | AT1G19610; PDF1.4; Predicted PR (pathogenesis-related) protein. Belongs to the plant defensin (PDF) family; 5e-4 |
| Both | ES36 | EST SSR | SS_CP_10_H11 | 1; 0.2 | Divergent | 3.22; 1.08 | 0.56; 0.49 | XM_002519072.1; Ricinus communis putative ring finger protein; 2e-10 | AT3G18930; RING/U-box superfamily protein; Functions in: zinc |

| | | | | | | | | | |
|---|---|---|---|---|---|---|---|---|---|
| | | | | | | | | | ion binding; 4e-4 |
| Popns | EC1496B | EST SSR | multiple | 1; 12.2 | Divergent | 1.76 | 0.50 | FJ594490.1; Gossypium hirsutum alpha-tubulin 10 mRNA; 0 | AT4G14960; TUA6; Tubulin alpha-6; isoform required for right handed helical growth; 0 |
| Popns | Ray2aB | indel | gi\|261923755\| | 1; 10.4 | Divergent | 1.59 | 0.50 | JF489914.1; Helianthus annuus cultivar cmsHA89 cycloidea-like protein (cyc2e) gene; 1e-53 | AT1G67260; TCP1; DNA binding and protein-protein interactions. Orthologue of Antirrhinum gene Cycloidea; 6e-23 |
| Popns | EC402 | EST indel | multiple | 8.1; 16.2 | Divergent | 1.56 | 0.48 | XM_002272259.2; Vitis vinifera 50S ribosomal protein L21; chloroplastic- | AT1G35680; Chloroplast ribosomal protein L21; RPL21C; |

| | | | | | | | | | |
|---|---|---|---|---|---|---|---|---|---|
| | | | | | | | | like; 5e-32 | Functions in: structural constituent of ribosome; RNA binding; Involved in: response to cold; translation; 5e-12 |
| Popns | EC733_260 | EST SSR | multiple | 3; 39.4 | Divergent | 2.49 | 0.54 | BT144679.1; Lotus japonicus clone JCVI-FLLj-6C23 unknown mRNA; 1e-90 | AT5G47030; ATP synthase subunit delta' mRNA; Functions in: hydrogen ion transporting ATP synthase activity; rotational mechanism; zinc ion binding; Involved in: response to salt |

| | | | | | | | | | |
|---|---|---|---|---|---|---|---|---|---|
| | | | | | | | | | stress; 2e-82 |
| Popns | E1M3_294 | AFLP | n/a | 8.1; 14.7 | Divergent | 2.23 | 0.52 | | |
| Popns | E1M7_91 | AFLP | n/a | 6; 0.5 | Divergent | 2.29 | 0.52 | | |
| Popns | E1M5_125 | AFLP | n/a | 8.1; 11.4 | Divergent | 1.56 | 0.49 | | |
| Popns | E8M5_186 | AFLP | n/a | 9; 7.1 | Divergent | 1.53 | 0.48 | | |
| Popns | E8M5_195 | AFLP | n/a | 10.1; 5.6 | Divergent | 1.50 | 0.48 | | |
| Popns | E1M3_210 | AFLP | n/a | unmapped | Divergent | 1.35 | 0.48 | | |
| Species | ES82 | EST SSR | SA_CP_07_A05 | 1; 41.5 | Divergent | 1.04 | 0.54 | AM451047; Vitis vinifera contig VV78X052823.4; 10 | AT2G27730; Functions in: copper ion binding; Involved in: photorespiration; 0.75 |
| Both | V45 | SSR | gi\|50363209\| | 9; 5.8 | Convergent | 2.37; 2.96 | 0.12; 0.09 | | |
| Both | ES4 | EST SSR | SA_CP_09_A12 | 10.1; 4.1 | Convergent | >5.00; 4.00 | 0.12; 0.10 | DQ336287; Vitis pseudoreticulata | AT4G08685; SAH7; Encodes a protein; |

| | | | | | | | | | |
|---|---|---|---|---|---|---|---|---|---|
| | | | | | | | | pollen-specific protein; 2e-55 | expressed in leaves; with similarity to pollen allergens; 1e-30 |
| Popns | S10 | SSR | gi\|50363186\| | 4; 11.5 | Convergent | 1.42 | 0.11 | | |
| Popns | E4M7_301 | AFLP | n/a | unmapped | Convergent | 1.17 | 0.09 | | |
| Species | S20 | SSR | gi\|50363191\| | 3; 24.3 | Convergent | 1.57 | 0.14 | | |

**Legend to Table S9.** Analysis indicates if selected marker was identified from a Bayescan analysis specifying species; populations (popns); or both these analyses. Marker type indicates the type of genetic marker. EST before the marker type indicates that it came from expressed sequence tags; otherwise markers were designed from anonymous DNA sequence. SSR indicates simple sequence repeat marker; indel indicates an insertion deletion polymorphism marker; and AFLP indicates amplified fragment length polymorphism. Source EST or sequence indicates publicly accessible sequence ID. Source names starting with “S” are from the *Senecio* expressed sequence tag database and source names starting with “gi” from the National Centre for Biotechnology Information (NCBI) sequence database. Only markers considered significantly divergent or convergent with $\log_{10}$ Bayes Factor values >1 are presented. Est. $F_{ST}$ are genetic differentiation values estimated according to the Bayesian genetic differentiation analysis. Best match genes and Ath. best match genes were identified as the minimum expect value gene entries identified by TBLASTX searching the NCBI nucleotide and *Arabidopsis*

*thaliana* databases, respectively. Additional summary gene information is provided when available. The search was performed on 11$^{th}$ Sep 2012.
